# Diversity of Reactive Astrogliosis in CNS Pathology: Heterogeneity or Plasticity?

**DOI:** 10.3389/fncel.2021.703810

**Published:** 2021-07-26

**Authors:** Aaron J. Moulson, Jordan W. Squair, Robin J. M. Franklin, Wolfram Tetzlaff, Peggy Assinck

**Affiliations:** ^1^Faculty of Medicine, University of British Columbia, Vancouver, BC, Canada; ^2^International Collaboration on Repair Discoveries (ICORD), Vancouver, BC, Canada; ^3^Department of Clinical Neuroscience, Faculty of Life Sciences, Center for Neuroprosthetics and Brain Mind Institute, École Polytechnique Fédérale de Lausanne (EPFL), NeuroRestore, Lausanne University Hospital (CHUV), University of Lausanne (UNIL), Lausanne, Switzerland; ^4^Wellcome Trust - MRC Cambridge Stem Cell Institute, University of Cambridge, Cambridge, United Kingdom; ^5^Department of Zoology, University of British Columbia, Vancouver, BC, Canada; ^6^Department of Surgery, University of British Columbia, Vancouver, BC, Canada; ^7^Centre for Regenerative Medicine, Institute for Regeneration and Repair, University of Edinburgh, Edinburgh, United Kingdom

**Keywords:** reactive astrocytes, heterogeneity, plasticity, single-cell RNA sequencing, ischemic stroke, CNS demyelination, traumatic brain injury (TBI), spinal cord injury (SCI)

## Abstract

Astrocytes are essential for the development and homeostatic maintenance of the central nervous system (CNS). They are also critical players in the CNS injury response during which they undergo a process referred to as “reactive astrogliosis.” Diversity in astrocyte morphology and gene expression, as revealed by transcriptional analysis, is well-recognized and has been reported in several CNS pathologies, including ischemic stroke, CNS demyelination, and traumatic injury. This diversity appears unique to the specific pathology, with significant variance across temporal, topographical, age, and sex-specific variables. Despite this, there is limited functional data corroborating this diversity. Furthermore, as reactive astrocytes display significant environmental-dependent plasticity and fate-mapping data on astrocyte subsets in the adult CNS is limited, it remains unclear whether this diversity represents heterogeneity or plasticity. As astrocytes are important for neuronal survival and CNS function post-injury, establishing to what extent this diversity reflects distinct established heterogeneous astrocyte subpopulations vs. environmentally dependent plasticity within established astrocyte subsets will be critical for guiding therapeutic development. To that end, we review the current state of knowledge on astrocyte diversity in the context of three representative CNS pathologies: ischemic stroke, demyelination, and traumatic injury, with the goal of identifying key limitations in our current knowledge and suggesting future areas of research needed to address them. We suggest that the majority of identified astrocyte diversity in CNS pathologies to date represents plasticity in response to dynamically changing post-injury environments as opposed to heterogeneity, an important consideration for the understanding of disease pathogenesis and the development of therapeutic interventions.

## Introduction

Astrocytes are critical for the functioning of the adult central nervous system (CNS) in health and disease with a myriad of well-documented roles encompassing the spectrum of physiologic functions from metabolic support to blood-brain-barrier (BBB) integrity to synapse regulation (for review see Volterra and Meldolesi, 2005; Abbott et al., 2006; Koehler et al., 2008; Sofroniew and Vinters, 2010a; Clarke and Barres, 2013; Bayraktar et al., 2015; Khakh and Sofroniew, 2015; Khakh and Deneen, 2019). There is growing consensus that astrocytes are highly plastic in response to environmental fluctuations, particularly in the dynamically changing environment of CNS pathological states. This has shifted our understanding of this ubiquitous glial cell population from that of a binary-population of fibrous and protoplasmic types to one with significant variation across multiple variables, including temporal, topographical, sex, and age, both within and across pathological states. Accordingly, there is significant interest in better understanding and further defining this potential diversity within the astrocyte population.

For the purposes of this review, we define diversity broadly as any distinguishable morphological, physiological, transcriptomic, proteomic, metabolic, or functional difference within the astrocyte population, whether transient or not. The development of technologies enabling detailed descriptions of these responses has led to an accumulation of evidence for diversity within the astrocyte population in the healthy CNS but also across many different models of disease/injury. Here, we briefly address the origin of astrocytes during development and what is currently known about the diversity of astrocytes in the healthy adult CNS prior to an in-depth exploration of diversity in CNS injury/disease, which is the focus of this review. Finally, we initiate a discussion on whether diversity should be sub-divided into more biologically meaningful categories, such as “plasticity” and “heterogeneity.” We propose that the use of these definitions provides a framework that will be important as more is discovered about astrocyte diversity in CNS pathologies with specific relevance for future therapeutic development.

## Astrocytes in Development and Astrocyte Diversity in Adult Homeostasis

### Astrocytes in the Developing CNS

At the foundation of our discussion on astrocyte diversity in the adult CNS is the multitude of studies on the developmental origin of astrocytes, which we only discuss in brief here (for more details see the following reviews: Bayraktar et al., 2015; Molofsky and Deneen, 2015). The majority of astrocytes originate from subventricular zone (SVZ) resident neuroepithelium-derived radial glial (RG) cells (Noctor et al., 2002; Anthony et al., 2004; Kriegstein and Alvarez-Buylla, 2009), with additional contributions from marginal zone progenitor cells in superficial cortical layers (Costa et al., 2007; Breunig et al., 2012). Importantly, embryonic astrogliogenesis accounts for only a fraction of adult astrocytes, as the majority of murine gliogenesis occurs postnatally (Bandeira et al., 2009) through the symmetric division of differentiated astrocytes (Ge et al., 2012). Direct transformation of RG cells is also a documented source of astrocytes (Merkle et al., 2004; Ghashghaei et al., 2007). Furthermore, NG2 glia (also referred to as oligodendrocyte progenitor cells—OPCs) have been reported to generate a distinct sub-type of ventral forebrain astrocytes (Zhu et al., 2008, 2011; Huang et al., 2014; Nishiyama et al., 2015). Once generated, astrocyte progenitors disperse radially from their site of origin within the confines of a single column leading to the establishment of a diverse population (Magavi et al., 2012; Gao et al., 2014). In the spinal cord, patterning of astrocyte progenitors is initiated by dorsoventral gradients of secreted molecules that facilitate radial organization. For example, three neural tube progenitor domains give rise to three spatially distinct ventral white matter (WM) astrocytes clusters (Hochstim et al., 2008) which are then likely influenced by a combination of intrinsic and extrinsic factors (for review, see Ben Haim and Rowitch, 2017). More fate-mapping analysis is required to establish the extent to which this developmentally established diversity persists into the adult CNS and contributes to the observed adult astrocyte diversity, both in the healthy CNS and in pathological states (e.g., Tsai et al., 2012).

### Diversity of Astrocytes in the Healthy Adult CNS

Beginning with Cajal’s descriptions of diverse morphologies amongst human and rodent astrocytes over 100 years ago, astrocyte diversity has been a recognized feature of the healthy adult CNS (Zhang and Barres, 2010; Boulay et al., 2017; Lin et al., 2017; Buosi et al., 2018; Matias et al., 2019). Early histologic description designated protoplasmic and fibrous astrocytes as unique subsets (Kölliker, 1889; Raff et al., 1984; Raff, 1989; Andriezen, 1893) based upon differences in location [WM vs. gray matter (GM)], cell body morphology, and interaction with neighboring neuronal structures (Bushong et al., 2002; Oberheim et al., 2012; Lundgaard et al., 2014). Transcriptional-based approaches have expanded upon this initial description (Cahoy et al., 2008; Batiuk et al., 2020; Bayraktar et al., 2020) with unique astrocytic gene profiles demonstrated across various brain regions (Chai et al., 2017; Morel et al., 2017; Duran et al., 2019; Bayraktar et al., 2020). Using fluorescence-assisted cell sorting (FACS) and immunohistochemical approaches, Lin et al. (2017) identified five distinct astrocyte populations in the mouse CNS, which displayed diverse synaptogenesis mechanisms. Similarly, using a transcription factor motif discovery approach, Lozzi et al. (2020) found region-specific astrocytic expression profiles in astrocyte populations from the olfactory bulb, hippocampus, cortex, and brainstem. Furthermore, astrocyte reporter mouse lines exposed molecular differences between different astrocyte populations within the adult cortex (Morel et al., 2019).

Importantly, these unique transcriptomic gene profiles are correlated with neural-circuit-based functional differences (Höft et al., 2014; Chai et al., 2017). Variable expression of key functional components in astrocytes has been noted, including glutamate receptors, transporter proteins, and ion channels (Matthias et al., 2003; Isokawa and McKhann, 2005; Olsen et al., 2007), as well as in calcium (Ca^2+^) signaling dynamics (Takata and Hirase, 2008), which is theorized to have a functional role in astrocyte-neuron communication (Bazargani and Attwell, 2016; Chai et al., 2017; Yu et al., 2018). Using multi-photon confocal imaging, Takata and Hirase (2008) demonstrated significant variance in astrocytic Ca^2+^ activity between cortical layer I and layers II/III. Diversity amongst astrocyte populations in different cortical layers has been identified in other studies as well (Lanjakornsiripan et al., 2018; Bayraktar et al., 2020). For example, single-cell RNA-seq (scRNA-seq) analysis identified five transcriptionally distinct clusters distributed amongst cortical layer I and III-V (Bayraktar et al., 2020) and quantification of astrocyte marker expression across cortical layers in the developing mouse brain revealed significant diversity across functionally distinct cortical areas (Batiuk et al., 2020).

Glial fibrillary acidic protein (GFAP)—a major component of intermediate filaments in astrocytes—is a widely used marker of astrocytes (Eng et al., 1971). Importantly, the basal level of GFAP in astrocytes in the healthy CNS is variable (Griemsmann et al., 2015; Ben Haim and Rowitch, 2017). For example, hippocampal astrocytes display higher GFAP expression than striatal astrocyte populations (Chai et al., 2017). Furthermore, GFAP expression is higher amongst spinal cord astrocytes compared to the brain (Yoon et al., 2017). Interestingly, astrocytic GFAP expression is modulated by various extrinsic stimuli, including global physical activity (Rodriguez et al., 2013), exposure to enriched environments (Rodriguez et al., 2013), and glucocorticoid treatment (O’Callaghan et al., 1991), suggesting that plasticity may play a role in shaping the regional diversity of GFAP expression observed in the healthy CNS. Interestingly, GFAP expression also fluctuates with circadian rhythms in the suprachiasmatic nucleus of the thalamus (Gerics et al., 2006). Also, GFAP expression amongst progenitor cells depends upon the developmental stage, highlighting more diversity in the expression of this marker (Cahoy et al., 2008; Kriegstein and Alvarez-Buylla, 2009; Roybon et al., 2013). While an important marker used to identify astrocytes, it is important to note that GFAP is not considered sufficient as an identifier of astrocyte populations, either in the healthy or injured CNS. A combination of multiple astrocyte markers is generally viewed as an improved approach [e.g., GFAP, aldehyde dehydrogenase-1 (Aldh1L1), and glutamine synthetase (GS); Serrano-Pozo et al., 2013]. Importantly, diversity in the expression of these additional markers is also seen (Anlauf and Derouiche, 2013; Waller et al., 2016). For example, diverse expression of Aldh1L1 amongst cortical astrocytes (Waller et al., 2016) and GS amongst entorhinal cortical astrocytes is observed (Anlauf and Derouiche, 2013).

As astrocytes are critical to the normal functioning of local neuronal populations (Chai et al., 2017; Matias et al., 2019), further characterization of the extent of this diversity (for review see Khakh and Sofroniew, 2015; Ben Haim and Rowitch, 2017; Khakh and Deneen, 2019), and the functional implications for neural circuit functioning (for review see, Nagai et al., 2021b) in the healthy CNS is vital. Furthermore, it is imperative to establish a baseline (Tsai et al., 2012; Batiuk et al., 2020; Bayraktar et al., 2020) against which identified diversity in models of CNS insult can be interpreted in order to develop targeted interventions aimed at manipulating aberrant and/or pro-pathogenic responses (Batiuk et al., 2020; Sofroniew, 2020).

## Diversity in the Context of Reactive Astrogliosis

### Defining Reactive Astrogliosis

Various terms have been used to describe the range of astrocytic responses to CNS insult and/or environmental perturbation (Eddleston and Mucke, 1993; Anderson et al., 2014; Pekny and Pekna, 2014; Sofroniew, 2015). In line with a recently published consensus statement (Escartin et al., 2021), we define “reactive astrogliosis” as the process by which astrocytes change in response to pathology. This can include changes in transcriptional regulation, or biochemical, morphological, metabolic, and physiological remodeling potentially associated with functional adaptation to the post-injury environment. Reactive astrogliosis was long viewed as homogenous and functionally passive, consisting of a stereotyped set of changes driving the conversion of homeostatic astrocytes to a distinct phenotype—the “reactive astrocyte” (Eddleston and Mucke, 1993; Anderson et al., 2014; Pekny and Pekna, 2014; Sofroniew, 2015). However, current evidence challenges this view, instead pointing to the existence of remarkable diversity in terms of morphology and transcriptional profile in varied CNS disease states (Hamby and Sofroniew, 2010; Zhang and Barres, 2010; Oberheim et al., 2012; Anderson et al., 2014; Schitine et al., 2015; Yoon et al., 2017; Zeisel et al., 2018; Masuda et al., 2019; Matias et al., 2019; Valori et al., 2019; Escartin et al., 2021). This begs the question of how extensive this diversity really is (Cahoy et al., 2008; Ståhlberg et al., 2011; Yoon et al., 2017; Batiuk et al., 2020; Bayraktar et al., 2020).

### Role of Astrocytes in CNS Disease

Reactive astrogliosis is observed in virtually all neurological conditions, including epilepsy (Steinhäuser et al., 2015), neoplastic disease (Priego et al., 2018; Heiland et al., 2019), demyelination (Woodruff and Franklin, 1999; Williams et al., 2007; Tassoni et al., 2019; Rawji et al., 2020), traumatic injury (Faulkner et al., 2004; Filous and Silver, 2016; Boghdadi et al., 2020a), neurodegeneration (Vargas et al., 2008; Ben Haim et al., 2015), and ischemic stroke (Zhao and Rempe, 2010; Zamanian et al., 2012; Rakers et al., 2019), as well as microbial CNS infections (Drögemüller et al., 2008; Soung and Klein, 2018; Geyer et al., 2019) and neurotoxin exposure (O’Callaghan et al., 2014; Wheeler et al., 2019). Reactive astrogliosis enables astrocytes to serve key roles in CNS pathological states, including metabolic support of vulnerable neurons, regulation of BBB permeability, remodeling of extracellular matrix (ECM), mobilizing progenitors, as well as immunomodulation, synaptic remodeling, and neurite outgrowth (Woodruff et al., 2004; Sofroniew and Vinters, 2010a; Anderson et al., 2014; Pekny and Pekna, 2014; Escartin et al., 2019, 2021). This process is regulated by a wide range of factors, both intrinsic and extrinsic to the CNS, and mediated through various cell surface receptors and intracellular signaling pathways (Sofroniew and Vinters, 2010a; Burda and Sofroniew, 2014; Sofroniew, 2015, 2020). Manipulation of these components in models of CNS injury/disease alters functional and histologic outcomes, demonstrating the importance of reactive astrogliosis and of understanding the extent and nature of its diversity (Brambilla et al., 2005, 2009; Okada et al., 2006; Herrmann et al., 2008; Haroon et al., 2011; Spence et al., 2011; Bonneh-Barkay et al., 2012; Wanner et al., 2013).

### Reactive Astrogliosis and the Aged CNS

Reactive astrogliosis is also a prominent feature of physiological aging in rodents, non-human primates, and humans (Nichols et al., 1993; Kanaan et al., 2010; Cerbai et al., 2012; Rodríguez et al., 2014; Jyothi et al., 2015; Robillard et al., 2016). Previous studies have noted regional diversity in astrocyte morphology, GFAP expression, and cellular density (Nichols et al., 1993; David et al., 1997; Hayakawa et al., 2007; Lynch et al., 2010; Cerbai et al., 2012; Geoffroy et al., 2016; Rodríguez et al., 2016). Aged astrocytes also demonstrate altered responses to CNS injury. For example, aged astrocytes display increased GFAP upregulation following SCI as compared to younger controls (Geoffroy et al., 2016), however, the functional implications of this remain unclear (Matias et al., 2019; Sutherland and Geoffroy, 2020).

Transcriptional approaches have once again greatly expanded our characterization of astrocyte diversity in the aged CNS (Soreq et al., 2017; Boisvert et al., 2018; Clarke et al., 2018). These studies have collectively revealed that aged astrocytes adopt a more pro-inflammatory phenotype (Orre et al., 2014), consistent with the concept that physiological aging is characterized by chronic low-level inflammation (i.e., “inflamm-aging”; Franceschi et al., 2000). Aged astrocytes from distinct brain regions display unique transcriptional profiles in both murine and human brains (Soreq et al., 2017; Boisvert et al., 2018; Clarke et al., 2018). Boisvert et al. (2018) performed RNA-seq analysis on 4 month and 2-year-old astrocyte-ribotag mice, demonstrating significant upregulation of pro-inflammatory and synapse elimination-related genes and decreased expression of cholesterol synthetic enzymes in the aged mice, with significant regional diversity (Boisvert et al., 2018). Expanding on this, Clarke et al. (2018) demonstrated regional diversity amongst aged astrocytes isolated (using the Bac-Trap method) from the hippocampus, striatum, and cortex, with upregulated genes related to astrocyte reactivity, immune response, and synapse elimination. Soreq et al. (2017) extended these findings to post-mortem human tissue of patients ranging in age from 16 to 102 years, revealing significant regional diversity of astrocyte-specific genes (Clarke et al., 2018). Characterization of the astrocyte secretome during the aging process may help validate many of these observed transcriptomic changes (Rawji et al., 2020). Intriguing questions remain as to the functional relevance of these age-related changes and the role of astrocyte diversity in the spatial propensity of various age-related disease processes (Ben Haim et al., 2015; Rodríguez et al., 2016; Matias et al., 2019). Furthermore, comparison of the transcriptional profiles of astrocytes across pathological states with those seen in the aged CNS may yield novel insights into disease pathogenesis, physiological aging, and the overlap between these states.

### Reactive Astrogliosis Diversity: Plasticity or Heterogeneity?

**FIGURE 1:**
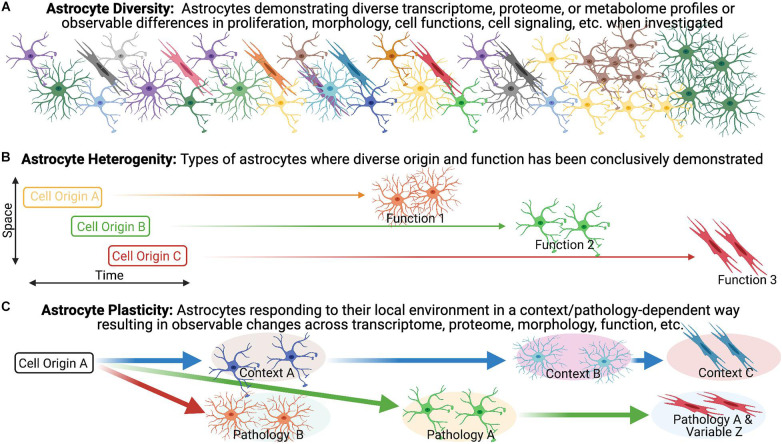
Astrocyte heterogeneity vs. astrocyte plasticity. **(A)** Evidence for diversity within the astrocyte population is becoming increasingly recognized and is particularly robust in the context of pathology/disease. **(B,C)** We highlight the importance of distinguishing astrocyte heterogeneity from astrocyte plasticity, as we define them, to direct our understanding of reactive astrogliosis and inform potential treatments.

We broadly define astrocyte diversity as any distinguishable morphological, physiological, transcriptomic, proteomic, metabolic, or functional difference within the astrocyte population, whether transient or not (Figure 1A). With this definition, and those that follow, we suggest that semantics are important, as a proper classification of diversity will likely lead to greater accuracy in the understanding of function and the directed development of therapeutic strategies. We therefore propose strict definitions to further classify reactive astrocyte diversity.

Heterogeneity has become a catch-all phrase that remains poorly defined, necessitating the need for clearer and unambiguous definitions. The term heterogeneity is derived from the Greek *heteros-* meaning “two, other, or different,” and the Latin *-genesis* meaning “origin or development” (Oxford English Dictionary). In distinguishing heterogeneity, we adhere to the definitions offered in a recent discussion of diversity in the oligodendrocyte lineage (Foerster et al., 2019), namely that heterogeneity implies distinct origin, as suggested in the definition, in combination with the demonstration of diverse functions (Figure 1B). In support of this, we look to other neural cell lineages. Firstly, heterogeneity amongst neurons is demonstrated by developmentally distinct neuronal subtypes with different transmission modes and firing patterns (i.e., function). A particularly relevant example of heterogeneity in the context of pathology lies within the microglia population. Dogma suggested that in mice, microglia progenitors arise at E7.5 from the yolk sac and then colonize the brain at E9.5 but mutant mice lead to the discovery that *Hoxb8* microglia (which express the *Hoxb8* transcription factor) represent a distinctive subpopulation of cells that are derived from a second wave which do not populate the brain until E12.5 (De et al., 2018). Although the non-*Hoxb8* microglia and *Hoxb8* microglia are very similar with few differentially expressed genes, they occupy distinct distributions in the post-natal mouse brain and demonstrate unique functional characteristics including their ability to participate in synaptic pruning and their response to injury. For example, the two microglia subpopulations are indistinguishable in their response to a stab wound injury in the acute phase (<30 min) but *Hoxb8* microglia demonstrated a greater tendency to accumulate at the injury epicenter at 7 days post-injury (dpi) compared to non-*Hoxb8* microglia (De et al., 2018). A similarly relevant example of heterogeneity in the context of pathology can be seen in the oligodendrocyte lineage. In the context of remyelination, OPCs arising from distinct ventral and dorsal domains during development have differential responses. For example, dorsally derived OPCs in the adult CNS demonstrate enhanced recruitment and differentiation into oligodendrocytes in response to demyelination as compared to their ventrally derived counter-parts (Crawford et al., 2016). Furthermore, dorsally derived OPCs demonstrate increased susceptibility to the age-associated differentiation impairment observed in the context of demyelination (Crawford et al., 2016). These findings illustrate the influence of heterogeneous populations on disease-associated variables (e.g., aging) in pathological settings (Crawford et al., 2016).

In the absence of pathology, one example of astrocyte heterogeneity (as we define it) is the demonstration that postnatal region-restricted spinal cord astrocytes have unique functions. In spinal cord development, spatially distinct astrocytes are specified through a homeodomain transcriptional code from positionally distinct progenitor populations (Hochstim et al., 2008) and spatially distinct domains remain stable throughout life in both mouse brain and spinal cord (Tsai et al., 2012). Specifically within the spinal cord, spatially distinct astrocytes with unique origins were shown to express postnatal region-specific genes and the ventral population plays a distinct role in sensorimotor circuit formation (Molofsky et al., 2014). This region-specific expression pattern of genes was further demonstrated across the cortical and subcortical adult mouse brain. Furthermore, astrocytes from these brain regions exhibited region-matched astrocyte to neuron communication specific to their ability to promote neurite growth and synaptic activity *in vitro* (Morel et al., 2017). Therefore, these spatially distinct astrocytes are a prime example of heterogeneity, as we define it, within the uninjured astrocyte population due to the direct evidence underlying their distinct origin and functions.

In contrast to our heterogeneity definition, plasticity would manifest as malleable morphological and/or phenotypic profiles among cells of a common origin in response to changing environmental conditions (Figure 1C). An illustration of this concept can be seen in the macrophage lineage. Monocyte differentiation into effector phenotypes occurs in accordance with local microenvironmental signals, accounting for the diverse macrophage effector functions in various tissues, and in response to different insults (Mosser and Edwards, 2008; Italiani and Boraschi, 2014; Lavin et al., 2014; Xue et al., 2014; Kuznetsova et al., 2020). Applied to the astrocyte lineage, plasticity would imply the CNS is populated with a homogenous population of astrocytes that undergo specialization at their final location as directed by local environmental features. For example, in the healthy postnatal CNS, the functional maturation of cortical astroglia is modified by the loss of neuronal glutaminergic signaling (Morel et al., 2014). In the adult healthy CNS, sonic hedgehog released from local neurons plays an active role in regulating both astrocyte function and the astrocytes’ molecular profile (Farmer et al., 2016) demonstrating that astrocytes can respond to cues from neurons that drive their properties/functions. We suggest that for distinct reactive astrocyte populations to be considered heterogeneous, definitive demonstration of distinct origins and functions need to be established to effectively exclude plasticity.

While we segregate plasticity and heterogeneity here for conceptual purposes, it is likely that there is a dynamic interplay between the two with varying contributions across multiple disease variables. An example of this can be seen in the oligodendrocyte lineage. While the diversity of myelin internode length appears to be a function of the axon characteristic and not oligodendrocyte diversity (i.e., plasticity; Chong et al., 2012; Tomassy et al., 2014), diverse oligodendrocytes isolated from either the spinal cord or cortex form myelin sheaths of different lengths when provided artificial microfiber as a substrate for myelination (Bechler and Byrne, 2015), suggesting at least a degree of intrinsic determination (i.e., heterogeneity; Crawford et al., 2016). Importantly, diversity in internode length is reduced when oligodendrocytes were cultured with dorsal root ganglion neurons or brain slices, implicating both intrinsic (i.e., heterogeneity) and extrinsic factors (i.e., plasticity) in determining the outcome. It is likely that a similar dynamic combination exists for astrocytes as well. Importantly, this is not just a semantic argument, as recognition of the contributions of plasticity to the observed diversity has the potential to reveal novel targets amendable to extrinsic manipulation *via* targeted therapeutic approaches across multiple aspects of disease pathology.

### Tools to Better Understand Cell Diversity in Reactive Astrocyte Populations

**FIGURE 2:**
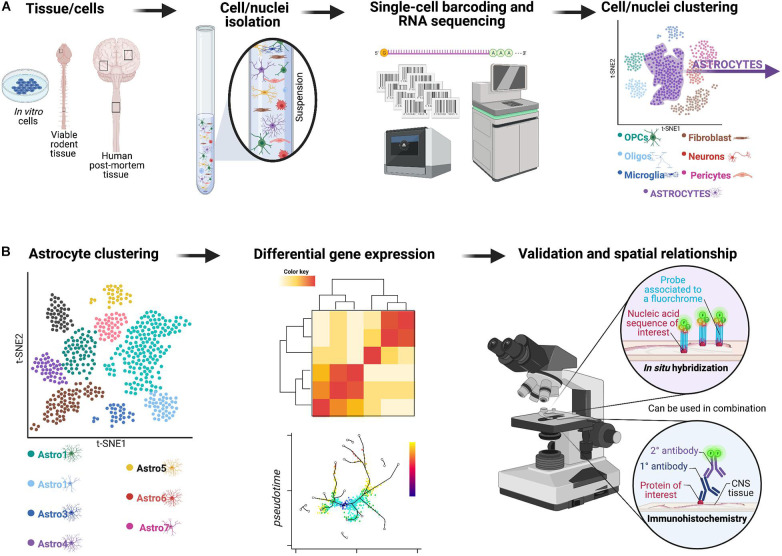
Overview of a potential workflow featuring droplet-based sc/snRNA-seq approaches to investigate astrocyte diversity across different CNS pathologies. **(A)** In the past few years, a technological revolution in RNA-sequencing technology has made it possible to profile the entire transcriptome of individual cells on a massive scale—a technique known as single-cell RNA-sequencing or scRNA-seq (Svensson et al., 2018). Initially, scRNA-seq relied on manual cell picking (Van Gelder et al., 1990; Eberwine et al., 1992) or FACS-based sorting (Ramsköld et al., 2012; Shalek et al., 2013). Innovative analyses revealed a surprising degree of transcriptional heterogeneity in seemingly homogenous cell populations. Subsequent advances in microfluidic instrumentation (Shalek et al., 2014; Treutlein et al., 2014) and droplet-based methods (Klein et al., 2015; Macosko et al., 2015) have since driven experimental costs down significantly to now permit sequencing of tens to hundreds of thousands of cells in a single experiment (Cao et al., 2017; Schaum et al., 2018). The rapid pace of methodological and computational progress has fostered initiatives to profile the mRNA landscape within every single cell of various model organisms (Cao et al., 2017; Schaum et al., 2018) and, ultimately, in humans (Rozenblatt-Rosen et al., 2017). This framework now enables comprehensive interrogation of the molecular etiology of human disease at single-cell resolution (Stubbington et al., 2017; Cheung et al., 2019). For example, single-cell transcriptomics offers an opportunity to elucidate how individual types of cells coordinate their activity to drive pathophysiological processes, and how cell type-specific responses might be targeted to treat disease. Indeed, in only the past few years, scRNA-seq has been applied to asthma (Braga et al., 2019), inflammatory bowel disease (Martin et al., 2019; Parikh et al., 2019; Smillie et al., 2019), obesity (Svensson et al., 2018), Alzheimer’s disease (Mathys et al., 2019), and TBI (Arneson et al., 2018), among other disorders. These technologies and analyses enable clustering of all viable cells/nuclei included in the original sample based on gene expression, and for example to identify astrocyte-like subpopulations can be isolated for further analyses. **(B)** The astrocyte-like subpopulations can be further clustered and examined using approaches, such as differential gene expression to yield important information about heterogeneous astrocyte populations.

scRNA-seq or single-nuclei RNA-seq (snRNA-seq) have enabled the direct quantification of single-cell or nuclei RNA complements at an increased resolution (Tang et al., 2009; Zeisel et al., 2018; Habib et al., 2020). Approaches, such as droplet-based sc/snRNA-seq approaches (Figure 2) have proven immensely powerful across various disease and injury models, revolutionizing our capacity for cellular characterization (Chen et al., 2018; Ding et al., 2020; Jäkel and Williams, 2020). Importantly, distinct clusters of astrocytes identified through these single-cell technologies need to then be validated to make sure results are not just noise and the spatial relationship needs to be re-established using *in situ* hybridization or immunohistochemistry alone or in combination. Use of these techniques also allow for analysis on human post-mortem tissue and *in vitro* culture systems (Grubman et al., 2019; Mathys et al., 2019) which has significantly enhanced our knowledge of species-specific differences in reactive astrogliosis, a critical hurdle for translation of pre-clinical findings in rodent model systems to human patients (Nichols et al., 1993; Oberheim et al., 2009, 2012; Soreq et al., 2017). When used alone these tools provide a powerful means of assessing astrocyte diversity but do not clearly distinguish between heterogeneity and plasticity unless they are combined with other complementary experiments, such as those looking at lineage-tracing. With that being said, recent papers highlight the potential of these single-cell technologies to yield information about lineage (Weinreb et al., 2020) as well as connectivity analysis (Clark et al., 2021) to be performed in high-throughput, with cell-type resolution. Furthermore, new techniques, such as sc-ATAC-seq will provide information about chromatin accessibility, which is likely to be an important determinant of astrocyte plasticity (Buenrostro et al., 2015). These new approaches will be influential in the task of determining whether reactive astrocyte clusters represent heterogeneity (with district origin and functions) or plasticity.

### The Spectrum of Reactive Astrogliosis

Similar to the healthy CNS, transcriptional analysis has revealed multiple clusters of reactive astrocytes in various models of CNS insult (Adams and Gallo, 2018; Tassoni et al., 2019; Yang et al., 2020). Various schemes have been proposed to categorize this diversity, including the classification of reactive astrocytes as either proliferative border-forming or non-proliferative in models of CNS trauma (Sofroniew, 2020). The most well-known is the categorization of astrocytes as neurotoxic “A1” or neuroprotective “A2.” In rodent models, intraparenchymal lipopolysaccharide (LPS)-injection induces a neurotoxic astrocyte phenotype stimulated by microglia-derived factors (Liddelow et al., 2017), whereas cerebral ischemia induces astrocytes to adopt what was later coined an “A2” phenotype that appeared to be neuroprotective (Zamanian et al., 2012). Astrocytes resembling what was later referred to as “A1” phenotypes have been identified in various disease states, including models of amyotrophic lateral sclerosis (ALS; Sun et al., 2015), Alzheimer’s disease (Sekar et al., 2015; Wu et al., 2019), prion disease (Smith et al., 2020), glioblastoma (Heiland et al., 2019), Parkinson’s disease (Chen et al., 2009), and Huntington’s disease (Diaz-Castro et al., 2019; Al-Dalahmah et al., 2020). While these “A1” vs. “A2” distinctions are useful, they also likely represent an oversimplification of the much larger continuum of reactive astrocyte states that are present in CNS pathologies. Indeed, the authors themselves stated that these two states were likely only a subset of many potential reactive states (Zamanian et al., 2012; Liddelow and Barres, 2017; Liddelow et al., 2017). In the recently published consensus statement, Escartin et al. (2021) highlight the shortcomings of using these binary divisions of reactive astrocytes, such as “A1” vs. “A2,” good vs. bad, or neurotoxic vs. neuroprotective and advocate for the assessment of multiple molecular and functional parameters moving forward (Escartin et al., 2021). Furthermore, a clear neurotoxic role for “A1” astrocytes is not always clearly demonstrated, highlighting the challenge with this dichotomous classification. For example, deletion of a subset of “A1” astrocytes accelerated neurodegenerative progression in a mouse model of prion disease (Hartmann et al., 2019), suggesting that these astrocytes states are considerably more nuanced. As will be discussed in the disease models below, transcriptional analysis has provided further evidence that astrocyte diversity exists along a spectrum of states likely driven by local microenvironments (Zhang and Barres, 2010; Anderson et al., 2014; Escartin et al., 2021). In this review, we still refer to “A1” vs. “A2” terminology for studies conducted in the past but adhere to the consensus put forward by Escartin et al. (2021) to avoid these terms in future research. Indeed, recent studies in models of ischemic stroke (Rakers et al., 2019; Androvic et al., 2020), demyelination (Yoon et al., 2017; Tassoni et al., 2019), and traumatic injury (Burda et al., 2016; Boghdadi et al., 2020b) have revealed substantial disease-specific, regional, and temporal reactive astrocyte diversity.

### Potential Variables Shaping Diversity in the Context of Reactive Gliosis

There are many variables potentially influencing the diversity of reactive astrogliosis. Here, we review the current state of knowledge on astrocyte diversity in the context of three representative and clinically relevant CNS pathologies: ischemic stroke, demyelination, and traumatic injury across multiple injury-associated variables (e.g., temporal, topographical, sex, and age). Plasticity can be conceptualized as the diversity in a response to environmental factors. In pathological states this includes features of the post-injury environment (e.g., cytokines, inflammatory cells, etc.). In contrast, heterogeneity manifests as diversity that results from differential origins (either developmental and adult-derived) and functionality. Importantly, plasticity and heterogeneity are not mutually exclusive in our model and elucidating this relationship as it manifests in various conditions of CNS pathology is critical to understanding the contributions of astrocytes in the injured CNS and how these responses can be manipulated. As our understanding of the extent of astrocyte diversity develops, it will become ever more important to integrate and compare astrocyte responses across pathological states to be able to interpret and apply the substantive datasets gleaned from single-cell transcriptomic approaches. To that end, we expand our discussion beyond the confines of a singular pathology and aim to highlight key limitations of our current knowledge, propose areas for future research, and discuss the relevance of this knowledge for therapeutic development. We specifically focus on the importance of differentiating the contributions of plasticity and heterogeneity to observed astrocyte diversity across multiple variables in CNS pathologies.

## Ischemic Stroke

Stroke is the primary cause of severe disability and a leading cause of death worldwide, associated with enormous socioeconomic burden (Cassidy and Cramer, 2017; Campbell et al., 2019; Campbell and Khatri, 2020). Accounting for 75–80% of all strokes (Cassidy and Cramer, 2017; Campbell et al., 2019; Campbell and Khatri, 2020), ischemic stroke results from the occlusion of a cerebral artery by a blood clot that either forms locally (i.e., thrombotic stroke) or more commonly, travels from another location, such as the heart or another proximal vessel (i.e., embolic stroke; Cassidy and Cramer, 2017; Campbell et al., 2019; Campbell and Khatri, 2020). Clinically, the extent of the resultant injury depends on several factors, including the severity and duration of ischemic injury and the quality of collateral blood flow to the affected perfusion territory (Bang et al., 2008; Maud et al., 2021). Despite recent advances in reperfusion techniques, therapeutic options remain limited and largely ineffective in attenuating the progressive neuronal loss and consequent functional impairment (Matei et al., 2021).

**FIGURE 3:**
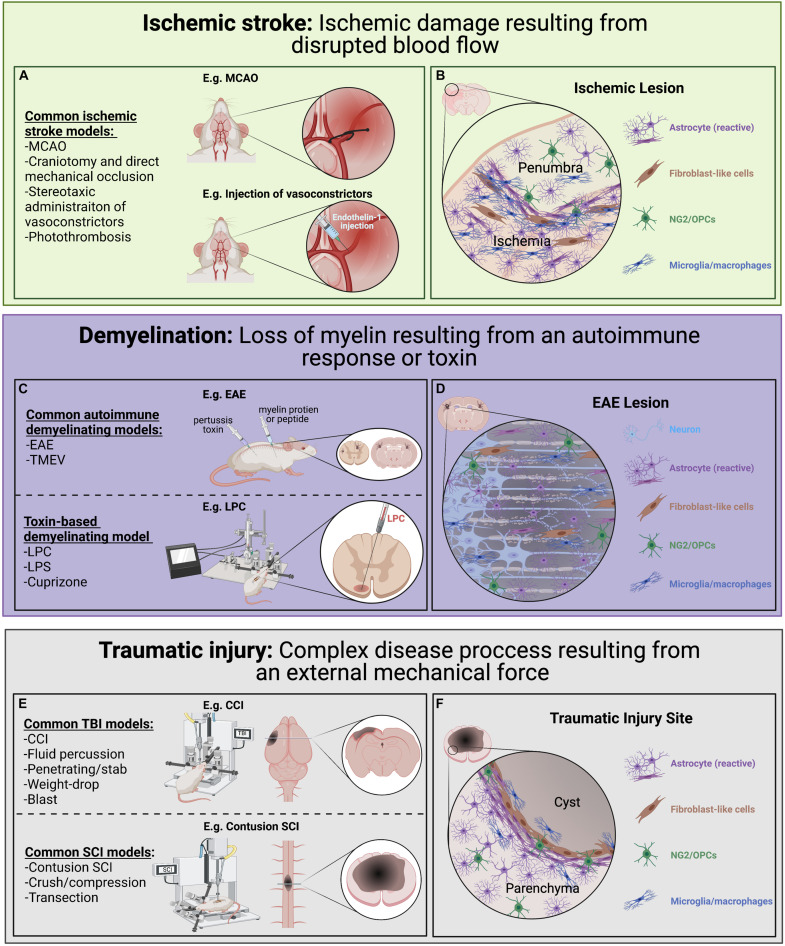
Pre-clinical models of ischemic stroke, CNS demyelination, and traumatic injury used to look at astrocyte diversity. Each of these models have advantages and disadvantages with regards to modeling human disease and induce diverse astrocyte injury responses. **(A)** Common ischemic stroke models. Ischemic stroke results from disrupted blood flow leading to ischemic damage, cell death and associated loss of function. Common animal models of ischemic stroke involve the transient or sustained blockade of normal blood flow to an area of the brain through occlusion of a blood vessel (e.g., MCAO). **(B)** A simplified illustration of an ischemic lesion where the ischemic area is predominately populated by immune cells and reactive astrocytes with a slow gradient toward an inner cluster of microglia/macrophages and surrounded by an outer layer of astrocytes. **(C)** Common demyelination models can be initiated through either an autoimmunity-based or toxin-based route, each highlighting different pathological features and chosen based on the research questions being pursued. **(D)** A simplified illustration of an EAE induced lesion where there is a loss of oligodendrocytes and their myelin sheaths (beige) within the lesion. Demyelination lesions are often filled with immune cells, including microglia/macrophages, and NG2/OPCs which contribute to repair. In the situation where remyelination does not take place, axon degeneration can result. **(E)** Common traumatic injury models focusing on different mechanical injuries applied to the brain and spinal cord, each with its own complex secondary injury cascade. **(F)** A simplified illustration of a typical CNS traumatic injury where a pronounced secondary injury cascade often leads to the loss of tissue (sometimes forming a fluid-filled cyst) at injury epicenter. Generally, there is an inner accumulation of fibroblast-like cells closest to epicenter surrounded by densely packed reactive astrocytes which have an important role in protecting the parenchyma tissue.

Various pre-clinical models of ischemic stroke have been employed (Figure 3A; Fluri et al., 2015; Rakers and Petzold, 2017; Sommer, 2017). Among these, the middle cerebral artery occlusion (MCAO) model is largely considered to most closely resemble human ischemic stroke (Longa et al., 1989; Rakers and Petzold, 2017; Sommer, 2017). Other frequently used models include direct mechanical occlusion of a cerebral vessel *via* clipping, ligation, or cauterization (Robinson et al., 1975; Chen et al., 1986; Brint et al., 1988; Hossmann, 2012), stereotactic administration of potent vasoconstrictors (e.g., endothelin-1) to induce vasospasm (Robinson and McCulloch, 1990; Sozmen et al., 2009; Roome et al., 2014; Fluri et al., 2015), and targeted activation of systemically administered photosensitive dye *via* transcranial illumination to induce localized thrombosis (i.e., the photothrombotic model; Watson et al., 1985; Kim et al., 2000; Kleinschnitz et al., 2008). Comparing tissue damage and astroglia responses across the range of pathology encompassed by these models will be informative. Another important consideration is the use of transient occlusive models, which mimic timely recanalization therapy (e.g., thrombolytics or endovascular thrombectomy; Sommer, 2017).

### Role of Astrocytes in Ischemic Stroke

The peri-infarct region following ischemic stroke (i.e., the zone immediately surrounding the ischemic lesion; Figure 3B), is commonly segregated into a monocyte/macrophage-dense inner region directly bordering the lesion and an astrocyte-rich outer region (Schroeter et al., 1995; Pekny and Nilsson, 2005; Gliem et al., 2015). The peri-infarct reactive astrocytes that populate this outer region secrete a variety of pro-inflammatory cytokines, chemokines, and matrix metalloproteinases that disrupt the BBB and recruit peripheral leukocytes, which are predominant contributors to secondary injury (Gliem et al., 2015; Yang and Rosenberg, 2015; Choudhury and Ding, 2016). Furthermore, astrocytes in this region demonstrate process elongation and polarization, as well as upregulation of several factors involved in ECM reorganization and reactive astrocyte clustering (Hirsch et al., 1994; Peng et al., 2013; Gliem et al., 2015). Using an RNA-seq approach in a MCAO rodent model, Rakers et al. (2019) demonstrated a significant upregulation of various neurotoxic genes associated with the “A2”-specific transcripts at 72 h post-stroke compared to control tissue. In total, >1,000 genes were differentially expressed after focal ischemia, including 38 transcription factors (Rakers et al., 2019), demonstrating the pronounced transcriptional changes that take place in response to ischemia. Importantly, many peri-infarct neurons survive the initial ischemic insult but undergo delayed degeneration due to progressive secondary damage to the surrounding spared tissue (Marchal et al., 1996; Liu et al., 2010; Chamorro et al., 2016). As astrocytes provide critical neuronal support and survive the ischemic insult in large numbers they represent an attractive target for therapeutic manipulation to promote, or at least mitigate, this neuronal loss and provide an increased substrate for repair (Zhao and Rempe, 2010; Liu and Chopp, 2016; Becerra-Calixto and Cardona-Gómez, 2017). As astrocytes are key players in both the pathogenesis of ischemic stroke and recovery process following insult, they are of significant interest. Clarification of the interplay between astrocyte plasticity and heterogeneity across multiple disease-associated variables (e.g., temporal, topographical, age-related, sex-related) promises to open novel therapeutic avenues aimed at mitigating stroke-associated morbidity and mortality.

### Astrocyte Diversity in Ischemic Stroke

Transcriptomic studies have revealed astrocyte diversity in models of ischemic stroke (Zamanian et al., 2012; Rakers et al., 2019; Boghdadi et al., 2020a). For example, ischemic insult induces differential expression of a host of genes in diverse subsets of astrocytes, including genes involved in neuroinflammation, apoptosis, and transepithelial migration of leukocytes (Zamanian et al., 2012) as well as cell division and migration (Rakers et al., 2019). Application of snRNA-seq provided refinement of this diversity in an endothelin-1-induced ischemic stroke model in marmosets, revealing 19 discrete reactive astrocyte subsets in the primary visual cortex (Boghdadi et al., 2020a). Interestingly, these subsets were noted to express a mixture of “A1” and “A2” genes (Boghdadi et al., 2020a), further highlighting the limited utility of applying binary categorization to what is most likely a nuanced spectrum of activation states. Interestingly, certain astrocyte subsets in the peri-infarct region expressed Nogo-A (Boghdadi et al., 2020a), well-known as a robust neurite outgrowth inhibitor (Chen et al., 2000; GrandPre et al., 2000; Kim et al., 2004; Schwab, 2004). Astrocyte diversity in models of ischemic stroke most likely represents predominant contribution of plasticity in response to the changing post-injury environment superimposed upon a background of heterogeneity, manifested in the post-natal brain as regionally specific astrocyte subsets with variable responses to ischemic insult. Elucidating the relative contributions of these processes to observed diversity along several disease-associated variables will require fate-mapping and functional interrogation of astrocyte subsets to reveal the prospective therapeutic potential.

### Potential for Astrocyte Plasticity Based on Disease-Associated Variables in Ischemic Stroke

In line with our definition, we propose that identified astrocyte diversity in ischemic stroke occurring along variables of time after injury (i.e., temporal), distance from lesion epicenter (i.e., topographical), age at onset, and sex of the individual, likely represent a greater contribution from plasticity than heterogeneity.

Astrocytes undergo dramatic morphological changes following ischemic insult that evolve over time (Lukaszevicz et al., 2002; Shannon et al., 2007; Benesova et al., 2009; Matyash and Kettenmann, 2009; Li et al., 2014). Using a murine photothrombosis model, Li et al. (2014) revealed increased GFAP expression by day 2 post-insult, with acquisition of stellate morphology and cellular hypertrophy by day 4, and dense astrocyte clustering by day 6. Interestingly, reactive astrocytes became less hypertrophic after day 6 post-insult with gradual lengthening of cellular processes by day 10, reflecting maturation of the reactive astrocyte clustering. Li et al. (2014) then went on to assess astrocyte proliferative dynamics, revealing a peak at day 3 post-insult, with a decline through day 14. Similarly, using an endothelin-1-induced vasospasm rat model, Mestriner et al. (2015) demonstrated significant increases at 30 days post-insult in astrocyte density and increased process ramification and length, as compared to uninjured controls. They also compared these dynamic changes across stroke types, using a time-matched post-hemorrhagic stroke model as comparison. Despite not revealing observable differences in several different GFAP-immunohistochemistry-based measurements, this cross-model comparison approach remains important as it enables isolation of intrinsic vs. extrinsic influences on astrocyte responses in the post-stroke brain (Mestriner et al., 2015). Use of higher resolution transcriptomic studies comparing reactive astrocyte responses over time and across different stroke types (e.g., ischemic, hemorrhagic) and vascular territories (e.g., middle cerebral artery, posterior cerebral artery, etc.) are likely to yield important information on reactive astrocyte diversity.

Astrocytic Ca^2+^ signaling dynamics also vary temporally in rodent models of ischemic stroke, in brain slice culture models of ischemia, and oxygen-glucose deprivation (OGD) models, both acutely and chronically (Duffy and MacVicar, 1996; Ding, 2013, 2014). For example, significant variability in latency to increased Ca^2+^ levels is observed in the initial minutes following ischemic onset in an OGD model (Duffy and MacVicar, 1996; Ding, 2013, 2014), suggesting that differing environmental factors may play a role. Furthermore, application of two-photon imaging in an *in vivo* photothrombosis murine model revealed reactive astrocyte subsets with dynamically changing amplitude and frequency of Ca^2+^ signaling, attributed to fluctuations in extracellular glutamate and GABA levels (Ding et al., 2009), in keeping with our definition of plasticity. Diversity in astrocytic Ca^2+^ signaling can also be seen outside of the acute post-injury period (Winship and Murphy, 2008; Choudhury and Ding, 2016). Using a murine photothrombotic model, Winship and Murphy (2008) demonstrated a progressive increase in magnitude of penumbral astrocytic Ca^2+^ signaling for 2 months post-insult, driven by stimulation of neural circuits *via* limb stimulation. Once again this represents modulation of astrocyte diversity in response to environmental manipulation (e.g., neural circuit stimulation), thus is offered to represent plasticity. Ca^2+^ signaling diversity may have important functional consequences for the functioning of local neural circuits, thus representing a significant parameter to further characterize. As neural circuit remodeling is an important mechanism of functional recovery post-stroke, manifested by the relative success of physical therapy in stroke patients over the sub-acute to chronic period, it will be a priority to optimize environmental variables that promote supportive astrocyte phenotypes. Further elucidation of astrocyte diversity, especially plasticity vs. heterogeneity, in this context is an important aspect of understanding neural circuit remodeling and functional recovery in the post-stroke brain.

Significant astrocyte diversity is also seen in rodent models of ischemic stroke with respect to distance from the lesion site (i.e., proximal topographical variation). For example, in a rat MCAO model, GFAP immunostaining, process volume, diameter, length, and branching points were increased in close proximity to the cortical infarct border zone compared to distances slightly further from the ischemic area (Wagner et al., 2013). Using a photothrombotic murine stroke model, Li et al. (2014) demonstrated an outwards gradation of astrocyte proliferation from the lesion core in parallel with higher densities of GFAP+ reactive astrocytes. After ischemia due to endothelin-1-induced vasospasm in the primary visual cortex of marmosets, astrocyte subsets were shown to differ in their expression of multiple reactive astrocyte markers, immunomodulatory genes, and various cytokine pathways (Boghdadi et al., 2020a), as well as transcriptional regulators and cell surface receptors involved in cell-matrix adhesion and migration (Dzwonek and Wilczynski, 2015; Boghdadi et al., 2020a). Interestingly, a number of neuronal genes were also upregulated, including Growth Associated Protein 43 (*Gap-43*), which is involved in post-infarct plasticity and expressed by neurons after axotomy (Skene and Willard, 1981; Tetzlaff et al., 1991; Frey et al., 2000; Hung et al., 2016). Topographical diversity in astrocyte Ca^2+^ signaling is also seen, with reduced magnitude amongst penumbral astrocytes compared to the lesion core (Winship and Murphy, 2008). Moreover, restricted astrocyte Ca^2+^ signaling has been shown following single vessel occlusion photothrombosis (Zheng et al., 2013). As most work done to date examines proximal changes near the site of ischemia (i.e., within a singular brain region), this is most likely reflective of plasticity in response to gradients of injury-associated factors (e.g., cytokines, inflammatory cells, blood-borne elements) radiating out from the epicenter. One notable caveat is the observation of extensive changes amongst astrocytes occurring over significant distances (i.e., between different brain regions) post-stroke. For example, in a MCAO rodent model, Rakers et al. (2019) demonstrated significant changes in gene expression in both the ipsilateral and contralateral hemispheres compared to non-injured control tissue. They demonstrated a 2- and 12-fold increase in *Gfap* and a 2- and 20-fold increase in vimentin (*Vim*) in the contralateral and ipsilateral hemispheres, respectively, compared to uninjured control tissue (Rakers et al., 2019). Diversity noted amongst such spatially separated regions in response to injury could represent a relatively greater contribution from heterogeneity as the astrocytes in these regions most likely have different origins. Despite this, plasticity is still likely a key driver of the observed diversity, albeit proportionally less than for changes observed in close proximity to the lesion.

As ischemic stroke is predominately a disease of older individuals, understanding age-related astrocyte diversity is critical for therapeutic development (Badan et al., 2003; Orre et al., 2014; Androvic et al., 2020). Physiologic aging enhances ischemia-induced astrocyte reactivity, resulting in exaggerated glial responses and accelerated formation of densely packed astrogliosis borders as compared to younger controls (Badan et al., 2003; Popa-Wagner and Badan, 2007). As this is likely to be a consequence of extrinsic/environmental factors, we propose that this age-related diversity represents plasticity, at least within the confines of a singular brain region. Using the MCAO model in 18 month old aged mice, Androvic et al. (2020) demonstrated a predominance of aged astrocytes toward a neurotoxic “A1” phenotype as compared to younger animals, which was associated with worse functional outcomes (Badan et al., 2003; Androvic et al., 2020). This neurotoxic predominance is thought to be largely driven by aged microglia, which tend to be more pro-inflammatory and reactive to insult (Orre et al., 2014; Androvic et al., 2020), consistent with plasticity in response to an altered environment. Using a targeted striatal infarction model in rats, Lively et al. (2011) demonstrated an age-related reduction in astrocyte-derived synaptic cleft-1 (SC1) expression, an ECM molecule associated with neural plasticity, a key aspect of recovery following stroke (Badan et al., 2003; Lively et al., 2011; Sohrabji et al., 2013; Cassidy and Cramer, 2017). Furthermore, ischemia-activated primary astrocyte cultures isolated from aged rats display reduced glutamate uptake compared to younger controls (Lewis et al., 2012). Impaired astrocyte-mediated buffering of glutamate levels in the post-stroke brain may contribute to the increased infarct volumes and worse functional outcomes seen in aged rodents following ischemic stroke (Popa-Wagner and Badan, 2007; Selvamani and Sohrabji, 2010), further suggesting an environmentally dependent effect. Further investigation with lineage tracing studies in the context of the aged CNS is an important area to elucidate potential contributions from heterogeneity to this observed diversity.

Although age-specific stroke incidence and mortality are higher in men, stroke-related morbidity is greater amongst women (Reeves et al., 2008; Petrea et al., 2009), making sex an important disease-associated variable to investigate astrocyte diversity. Adult female rodents demonstrate smaller infarct volumes than age-matched males (Hall et al., 1991; Alkayed et al., 1998; Manwani et al., 2011; Selvamani et al., 2014), an effect which reverses with advancing age (Manwani et al., 2013) suggesting that the aged female brain is more susceptible to ischemic insult than its male counterpart (Chisholm and Sohrabji, 2016; McCullough et al., 2016). Importantly, these gross differences are underlain by sex-specific diversity amongst cellular populations, including astrocytes. Using a mouse MCAO model, Morrison and Filosa (2016) demonstrated sex-specific differences in frequency of astrocytic Ca^2+^ elevations as well as a more robust reactive astrocytic response in male mice as compared to age-matched females. Sex hormones appear to be a key player in this observed difference. Indeed, astrocyte-derived estradiol conveys neuroprotective and anti-inflammatory effects in rodent models of global ischemia (Zhang et al., 2014). Furthermore, cultured astrocytes isolated from female rodents are more resistant to *in vitro* ischemic insult and glucose deprivation, mediated in part by increased P450 expression and aromatase activity compared to male astrocytes, thus altering estrogen levels in the cells (Liu et al., 2007; Liu et al., 2008). Importantly, ischemia induces astrocyte-specific aromatase expression and activity *in vivo* as well (Carswell et al., 2005). Consistent with this, live imaging studies in a unilateral MCAO rodent model demonstrated significantly increased estrogen-dependent GFAP expression in adult female mice compared to adult males (Cordeau et al., 2008) and estrogen induces astrocytic expression of glutamate transporters GLT-1 and GLAST (Pawlak et al., 2005; Lee et al., 2009), suggesting that female astrocytes *in vivo* may be more effective at glutamate clearance than their male counterparts. Considering that much of this observed diversity is thought to be driven by sex hormones (e.g., estrogen, progesterone), we suggest that this astrocyte diversity is predominantly representative of plasticity, although we cannot exclude the potential of heterogeneity also being a contributing factor (McCullough et al., 2016). This is particularly interesting given the notable sex-specific difference in clinical outcomes (Reeves et al., 2008; Petrea et al., 2009) and the potential for modulation of hormone-responsive signaling pathways as a means of conveying neuroprotection, which are commonly targeted by therapeutics for malignancies (e.g., tamoxifen, trastuzumab; Jordan, 2003; Cameron et al., 2017; Shagufta and Ahmad, 2018; Xu et al., 2019). Adaptation of these therapeutics for the modulation of astrocytic responses in the post-stroke brain may be a viable approach for neuroprotection and/or post-stroke recovery. This requires detailed characterization of the extent of astrocyte diversity present following ischemic stroke, the functional implications of that diversity with respect to neural survival and function, and the identification of potentially malleable targets for therapeutics.

### Potential for Astrocyte Heterogeneity Based on Disease-Associated Variables in Ischemic Stroke

In contrast to plasticity, heterogeneity as a contributor to astrocyte diversity in ischemic stroke represents potential diversity that exists amongst astrocyte populations with established differences in origin and function. According to this definition, we propose that this would be diversity observed between regionally specific astrocyte populations (i.e., diversity across brain regions). In the context of ischemic stroke, this could be seen as diversity amongst astrocyte responses in diverse brain regions following a similar injury (i.e., infarction in different vascular territories). Mestriner et al. (2015) used endothelin-1-induced vasospasm rat model and compared ischemic lesions in the sensorimotor cortex and dorsolateral striatum and noted variation at 30 days post-insult in terms of GFAP-immunohistochemistry based measurements including cellular optimal density and primary process length (Mestriner et al., 2015). Using a MCAO murine model, Lukaszevicz et al. (2002) demonstrated differential functional responses of protoplasmic and fibrous astrocytes (thus effectively comparing WM and GM lesions), including differences in morphological response and cell death (Lukaszevicz et al., 2002), consistent with demonstration of differential ischemic sensitivities amongst astrocyte subsets in models of ischemia-reperfusion injury (Shannon et al., 2007). Importantly, it remains unclear whether protoplasmic and fibrous astrocytes have distinct origins considering that both can be derived from the postnatal SVZ during corticogenesis (Levison and Goldman, 1993; Parnavelas, 1999; Marshall and Goldman, 2002). Lineage tracing has shown that SVZ-derived multipotent neural stem cells (NSC) have the ability to migrate and contribute to the reactive astrocyte population in the context of multiple stroke models (Faiz et al., 2015) but it is unclear how different their function is from other reactive astrocytes in the region. Further direct lineage tracing experiments are required to establish these populations as truly distinct from an origin and functional perspective, and thus fitting of our offered definition of heterogeneity.

Due to limited high-quality fate-mapping studies, there is a scarcity of data on the extent of astrocyte heterogeneity in ischemic stroke. Furthermore, most data accumulated to date focus on astrocyte diversity as a function of changing environmental parameters, and therefore represent plasticity. Ischemic stroke has the potential to affect multiple vascular territories, and thus diverse populations of astrocytes. Determination of the impact of regionally specific astrocyte diversity to various post-stroke outcome measures is of great interest, both clinically and for understanding stroke pathophysiology. For example, hemorrhagic transformation of infarcted tissue (i.e., hemorrhage developing within infarcted tissue) is a devastating post-stroke complication caused by disruption of the BBB (Warach and Latour, 2004; Khatri et al., 2012; Sussman and Connolly, 2013), of which astrocytes are a key component. Identification of malleable aspects of astrocyte diversity that alter susceptibility of neurovascular degradation may be a viable approach to reduce occurrence in the post-stroke population. To that end, expanded study of reactive astrocyte diversity in ischemic stroke models affecting different vascular territories (e.g., middle cerebral artery, posterior cerebral artery, etc.) using combined fate-mapping and functional assays is warranted.

## CNS Demyelination

The most common demyelinating disorder is multiple sclerosis (MS), a complex immune disease characterized by inflammation, primary demyelination (loss of myelin from an intact axon), and neuronal/axonal damage/degeneration (Molina-Gonzalez and Miron, 2019; Rawji et al., 2020). Various pre-clinical models have been employed to replicate the complexity of the MS disease process (Figure 3C; Mix et al., 2010; Baker and Amor, 2015; Lassmann and Bradl, 2017; Baecher-Allan et al., 2018). One of the main models is experimental autoimmune encephalitis (EAE; Constantinescu et al., 2011; Ben-Nun et al., 2014), which involves the administration of myelin peptides or CNS homogenate to induce an autoimmune-mediated demyelinating insult to the rodent CNS (Denic et al., 2011; van der Star et al., 2012; Baker and Amor, 2015). While EAE models the immunopathogenesis of acute MS lesions and has been instrumental to discover many disease modifying treatments (Plemel et al., 2017), it is of limited value for assessing the neurobiological aspects of remyelination (Owens, 2006; Haanstra et al., 2015; Procaccini et al., 2015; Plemel et al., 2017; Stimmer et al., 2018). Another immune-mediated model of MS is the use of Theiler’s murine encephalomyelitis virus (TMEV) to induce spinal cord demyelinated lesions (Dal Canto and Lipton, 1977; Owens, 2006; Denic et al., 2011; McCarthy et al., 2012). Toxin-based models of demyelination have also been used widely in pre-clinical studies on neurobiological aspects of demyelinating disease. Most commonly employed are LPS (Liddelow et al., 2017), cuprizone (Praet et al., 2014), lysophosphatidylcholine (lysolecithin; LPC; Blakemore and Franklin, 2008; Lassmann and Bradl, 2017), and ethidium bromide (EB) models (Woodruff and Franklin, 1999; McMurran et al., 2019). LPS is a model of neuroinflammation initiated by the injection of a bacterial endotoxin and results in neurotoxic astrocyte phenotype (Liddelow et al., 2017). Cuprizone is a copper chelating agent that is administered orally and induces acute CNS demyelination, particularly in the corpus callosum and cerebellar peduncles (Praet et al., 2014). LPC by contrast, is focally injected into white matter tracts, inducing damage to the myelin sheaths (Blakemore and Franklin, 2008; Lassmann and Bradl, 2017) and acting as a chemoattractant for monocytes, thus triggering a focal inflammatory response (Blakemore and Franklin, 2008; Lassmann and Bradl, 2017). Many toxin-based demyelination models represent a simpler system with predictable and reproducible spatiotemporal patterns to study the remyelination process (McMurran et al., 2019) and have been used as tools to highlight both the permissive and inhibitory roles of astrocytes (reviewed by Rawji et al., 2020).

### Role of Astrocytes in CNS Demyelination

Astrocytes are central to the demyelination response and the success of remyelination (Molina-Gonzalez and Miron, 2019; Rawji et al., 2020), as they release anti-inflammatory cytokines, proteins that modulate myelin regeneration (remyelination), actively maintain extracellular ionic and neurotransmitter concentrations, and provide critical neuronal support (Hinks and Franklin, 1999; Jessen et al., 2015; Liddelow et al., 2017). Astrocytes also release pro-inflammatory cytokines, promote BBB permeability, and inhibit OPC maturation (Sisková et al., 2006; van Horssen et al., 2007; Lau et al., 2013; Stoffels et al., 2013). These contrasting functions are thought to represent changing functional roles at different stages of the remyelination process (reviewed in Rawji et al., 2020). Importantly, astrocyte responses are often dependent on the type of lesion (Rao et al., 2019) where acute active lesions are filled with immune cells and hypertrophic astrocytes with increased GFAP expression and an associated upregulation of proinflammatory chemokines and cytokines (Williams et al., 2007; Franklin and Goldman, 2015). Alternatively, inactive lesions (Figure 3D) have less inflammation and the astrocytes typically are more densely packed with long thick processes in close proximity to extensively demyelinated axons (Franklin and Goldman, 2015). In the rodent cuprizone model, astrocytes have been shown to regulate the recruitment of microglia which then facilitates myelin debris removal, an imperative step for subsequent repair (Skripuletz et al., 2013). Astrocytes also play an important role in influencing the balance of OPC-derived oligodendrocyte vs. OPC-derived Schwann cell-mediated CNS remyelination (reviewed by Chen et al., 2021). The ability of astrocytes to take on different roles may reflect distinct sub-populations or dynamic changes in malleable phenotypes driven by spatiotemporal environmental fluctuations (e.g., microglia-derived factors, cytokines; Cerbai et al., 2012; Horstmann et al., 2016). The relative contributions of heterogeneity and plasticity to these dynamically changing roles remains to be elucidated, as well as the potential for manipulation of astrocytes to phenotypes that support a pro-remyelination lesion environment.

### Astrocyte Diversity in CNS Demyelination

Similar to other CNS pathologies, transcriptional evidence has expanded our understanding of astrocyte diversity in CNS demyelination, including the use of bulk transcriptional analysis (Rothhammer et al., 2016, 2018; Itoh et al., 2018; Chao et al., 2019; Wheeler et al., 2019). For example, RNA-seq analysis of EAE and MS tissue identified several clusters of astrocytes with differential expression of *S100b*, *Gja1*, *Aldh1l1*, *Gfap*, and *Aqp4*, consistent with a spectrum of astrocyte transcriptional states in EAE (Wheeler et al., 2019). Furthermore, astrocytes in EAE and MS tissue were demonstrated to have variable expression of the transcription factor *Nrf2* and *Mafg* and *Mat2a* signaling, leading to DNA methylation and increased CNS pathology (Wheeler et al., 2020). These data identify epigenetic modifiers as potential therapeutic candidates to modulate pathology in MS. Importantly, as discussed below, there is a need to separate plasticity and heterogeneity in models of CNS demyelination to improve our understanding of factors that promote a pro-remyelination lesion environment and eventually guide therapeutic development. To that end, we propose here a framework for making that distinction using current evidence for astrocyte diversity in models of CNS demyelination as an example.

### Potential for Astrocyte Plasticity Based on Disease-Associated Variables in CNS Demyelination

We argue that astrocyte diversity across variables, such as disease stage (early or late disease progression), lesion status (status of demyelination or remyelination), age at disease onset, and sex of the individual are examples where plasticity is the predominant contributor to astrocyte diversity. In comparison to other CNS pathologies, MS and EAE have extensive spatiotemporal lesion variability and extensive immunological activity, complicating the interrogation of astrocyte diversity. For example, lesions present in the same individual at a given time can be at different stages (i.e., active, chronic active, inactive, remyelinated, etc.; Rao et al., 2019) and in different CNS regions (spanning WM and GM). Furthermore, analogous lesions can be present in individuals of different age and/or sex, complicating comparisons between spatiotemporally similar lesions. Given those complexities, we highlight the challenge in distinguishing plasticity and heterogeneity without clear fate-mapping studies to establish a baseline in regionally and temporally disparate CNS regions against which comparison of variability can be assessed.

Temporal evolution of astrocyte diversity in inflammatory demyelinated lesions has been noted. For example, in EAE models astrocyte cholesterol gene expression decreases late in the disease course in both the spinal cord and optic nerve (Itoh et al., 2018; Tassoni et al., 2019). Furthermore, astrocytic major histocompatibility complex-II (MHC-II) expression is markedly increased in early EAE brain and spinal cord lesions followed by a late decrease (Itoh et al., 2018), while optic nerve astrocytes in EAE optic neuritis show an early increase in MHC-II expression that persists through the disease course (Clarkson et al., 2015; Tassoni et al., 2019). Due to the role of MHC-II in immunomodulation, it is tempting to speculate about the functional relevance of this diversity, compounded by the fact that optic neuritis is commonly the initial presentation of MS in patients (Polman et al., 2005; Montalban et al., 2010; Amato et al., 2018). Diversity along the temporal component most likely represents response to a dynamic lesion environment, and thus plasticity. Despite this, it remains important to establish a baseline of heterogeneity, especially between CNS regions, as well as identify newly generated astrocytes in the context of demyelination injury as a potential source of heterogeneity.

Astrocyte diversity is also noted across lesion types in MS models (Rao et al., 2019). For example, acute lesions display hypertrophic astrocytes with increased expression of GFAP and several pro-inflammatory cytokines, chemokines, and remyelination-associated molecules (Woodruff et al., 2004; Williams et al., 2007; Franklin and Goldman, 2015; Rawji et al., 2020). By contrast, inactive lesions contain transcriptionally inactive astrocytes with small cell bodies and long filamentous processes that contribute to an increased density of reactive astrocytes (Franklin and Goldman, 2015; Ludwin et al., 2016). For example, acute lesions demonstrate reduced astrocytic connexin-43 (CX43) expression and disrupted CX43/CX47-mediated astrocyte-to-oligodendrocyte connections (Masaki et al., 2013). Loss of CX43 expression is associated with progressive MS disease, oligodendrocyte pathology, and astrocyte degeneration (Masaki et al., 2013) and patients with reduced CX43 expression have a more rapid clinical disease progression (Masaki et al., 2013). Furthermore, astrocytic chitinase 3-like 1 (CHI3L1) expression, associated with chronic inflammation and neurotoxicity (Cantó et al., 2012; Matute-Blanch et al., 2020), is observed in chronic MS lesions but absent from other lesion types (Cantó et al., 2012). One potential driver of this diversity is microglial-derived factors (e.g., activin-A), which have been shown to modulate astrocyte activation in demyelinated lesions (Miron et al., 2013; Rawji et al., 2020), further consistent with plasticity as a predominant contributor.

Age is an important factor in the pathogenesis of MS, with clinical disease onset rare after the age of 50 (Tremlett et al., 2010; Amato et al., 2018; Baecher-Allan et al., 2018). Furthermore, age at onset appears to be a key prognostic factor for MS patients (Tremlett et al., 2010; Amato et al., 2018). Progressive MS is also correlated with increasing age (Koch et al., 2009; Tutuncu et al., 2013). Despite the multitude of factors that may be contributing to this observation, including age-related changes in hormones, immune function, neural and non-neural cell populations, it is tempting to speculate regarding the potential contribution of age-related diversity in astrocyte subpopulations to this disease susceptibility. Aged-related astrocyte diversity observed is thought to be at least in part secondary to the increased pro-inflammatory function of aged microglia (Kanaan et al., 2010; Cerbai et al., 2012; Jyothi et al., 2015), thus likely representing plasticity. Aged astrocytes are more reactive and respond to demyelination with a more pronounced cellular hypertrophy (Robillard et al., 2016; Boisvert et al., 2018; Clarke et al., 2018). Aged astrocytes were also demonstrated to have decreased expression of key cholesterol synthetic enzymes (Boisvert et al., 2018; Clarke et al., 2018), which may contribute to the reduced success of remyelination with age (Shields et al., 2000; van Wijngaarden and Franklin, 2013). Further study will help to elucidate the impact of age-related astrocytic changes to remyelination success, either directly through immunomodulatory effects or indirectly through interactions with other neural cell lineages populating the lesions, as well as reveal astrocyte features potentially amendable to therapeutic targeting.

MS affects 2–3 times more women than men (Whitacre et al., 1999), but men are more likely to have disease progression (Savettieri et al., 2004; Koch et al., 2009; Tomassini and Pozzilli, 2009; Shirani et al., 2012). Sex-specific diversity of astrocytes is notable in models of auto-immune demyelination (Chowen et al., 2017), reflecting this clinical observation. In EAE optic neuritis, optic nerve astrocytes in female mice display more robustly increased *C3* expression (a component of the pro-inflammatory complement cascade; Stevens et al., 2007) and dampened upregulation of thombospondin-1 (*Thbs1*) when compared to their male counterparts (Tassoni et al., 2019), consistent with a more pro-inflammatory environment. Importantly, this is correlated with exaggerated retinal ganglion cell (RGC) and axonal loss in female mice (Tassoni et al., 2019), with a significant negative correlation between astrocytic expression of *C3* and RGC density (Liddelow et al., 2017; Tassoni et al., 2019). This is particularly interesting given the female predominance of clinical MS, as well as the frequency of optic neuritis as a presentation in female MS patients (Voskuhl and Gold, 2012; Baecher-Allan et al., 2018; Voskuhl et al., 2018). Importantly, these sex-specific differences were not observed in spinal cord astrocytes in the same model (Tassoni et al., 2019), highlighting notable regional diversity (Itoh et al., 2018). Further investigation to differentiate plasticity (e.g., hormone effects) from heterogeneity and to elucidate the functional relevance of this observed sex-specific diversity are important goals for future research (Chowen et al., 2017; Gilli et al., 2020).

### Potential for Astrocyte Heterogeneity Based on Disease-Associated Variables in CNS Demyelination

In contrast to plasticity, we propose that astrocyte heterogeneity in models of CNS demyelination likely manifests as differences in regionally specific astrocyte populations in disparate CNS regions (e.g., brain regions, spinal cord, etc.). For example, scRNA-seq analysis of isolated murine astrocytes after EAE revealed distinct expression profiles for spinal cord, cerebellar, and hippocampal astrocytes (Itoh et al., 2018; Tassoni et al., 2019). Cholesterol synthesis pathways were significantly downregulated in WM-rich regions (e.g., spinal cord, cerebellum, optic nerve) due to altered ApoE-mediated cholesterol transport (Itoh et al., 2018; Tassoni et al., 2019). This is consistent with the downregulation of cholesterol synthesis observed in chronic demyelinated lesions in mouse MS models (Boisvert et al., 2018; Clarke et al., 2018), thought to be detrimental for remyelination given the lipid-rich nature of myelin sheaths (Itoh et al., 2018; Rawji et al., 2020). There are also notable differences between astrocytes that populate subcortical WM and GM lesions (Albert et al., 2007; Prins et al., 2015). Combined snRNA-seq and transcriptomic lesion mapping in MS tissue enabled mapping of dysregulated genes to either GM or WM astrocyte subpopulations (Schirmer et al., 2019) revealing that GM astrocytes in cortical lesions (i.e., GPC5+/SLC1A2+ cells) had decreased expression of genes involving glutamate and K^+^ homeostasis, whereas WM astrocytes in subcortical lesions (i.e., CD44+/LINC01088+ cells) displayed upregulated genes including *GFAP*, the transcription factors *BCL6* and *FOS*, and endothelin receptor B (Schirmer et al., 2019). Spinal cord astrocytes in LPC focal demyelination models were shown to have increased GFAP expression as compared to the cerebral cortex (Yoon et al., 2017). Molecular pathway analysis identified the antigen-presentation and interferon signaling pathways as specifically enriched in spinal cord and cerebellar astrocytes (Itoh et al., 2018). Antigen presentation by astrocytes has been previously shown in EAE models (Cornet et al., 2000; Constantinescu et al., 2005) and thought to be functionally important in astrocyte-mediated immunomodulation (Rawji et al., 2016, 2020). Regional diversity can also be seen in EAE optic neuritis models (Horstmann et al., 2016; Nolan et al., 2018; Tassoni et al., 2019). scRNA-seq revealed astrocyte-specific upregulation of the complement cascade and *Thbs1*, a gene involved in RGC synaptic plasticity and visual recovery following demyelination (Tassoni et al., 2019). Astrocytic *Thbs1* expression peaks early in disease (Tassoni et al., 2019), consistent with early astrocyte regulation of neural plasticity in demyelinated visual circuits. Optic nerve astrocytes also displayed reduced expression of cholesterol synthetic genes and increased expression of antigen presentation genes (Tassoni et al., 2019), similar to that seen in spinal cord EAE lesions (Itoh et al., 2018). Diversity in gene expression was also noted between optic nerve and retinal astrocytes (Tassoni et al., 2019).

Regional diversity in EAE and MS is particularly interesting considering the multifocal nature of the disease (Baecher-Allan et al., 2018; Rawji et al., 2020). One can speculate that regional differences in astrocyte and other glial cell populations create an environment (e.g., ECM, cytokines, chemokines, etc.) more conducive to immune-mediated demyelination and/or for remyelination success. Astrocytes are known to exhibit regional specific responses to T-cell cytokines, leading to regionally specific neuroinflammatory responses in the hindbrain and spinal cord (Williams et al., 2020), suggesting that diverse astrocyte features may contribute to the anatomical propensity for demyelinated lesions to occur in certain locations (e.g., periventricular WM, spinal cord, optic nerve). Expression of pattern recognition receptors (PRRs) and interferon-induced genes, both at a basal level and in response to IFN induction, were higher in cerebellar than cortical astrocytes (Daniels et al., 2017), consistent with regionally specific astrocytic innate immune responses. Moreover, activation of the unfolded protein response alters the astrocytic secretome, generating a unique reactivity state and impairing synaptogenic function *in vitro* (Smith et al., 2020). Understanding the interplay between these environmental features and astrocytes is likely a fruitful area for therapeutic targeting, given the multitude of roles that astrocytes have in CNS demyelination. For example, GM lesions remyelinate more efficiently compared to WM (Albert et al., 2007; Bai et al., 2016; Strijbis et al., 2017), which could reflect, at least in part, contributions of regional specific astrocyte diversity. This will require detailed fate-mapping studies in models of CNS demyelination across lesion types, brain regions, and throughout the natural history of the condition.

## Traumatic Injury

Traumatic CNS injuries remain the major causes of disability, premature death, and long-term neuropsychiatric impairment (Fitzharris et al., 2014; Gbd, 2016; National Spinal Cord Injury Statistical Center, 2021). Traumatic spinal cord injury (SCI) results from the application of an external force on the spine (e.g., motor vehicle accident, fall, sports-related injury, violent injury) damaging the underlying neural tissue in a variable manner (Ahuja et al., 2017; National Spinal Cord Injury Statistical Center, 2021). The primary insult results in massive damage to neural cells and triggers a complex cascade of secondary injury mechanisms that culminate in neuronal and glial cell death, ischemic injury, and inflammation (Alizadeh et al., 2019). This is generally followed by significant reorganization of spinal cord structure, including the formation of a densely packed astrogliosis border surrounding a cystic cavity at the lesion site (Sofroniew, 2009; Adams and Gallo, 2018; Alizadeh et al., 2019; Tran et al., 2021). Traumatic brain injury (TBI) is highly variable mechanistically with a wide range of causative insults and severities. Moreover, TBI has a complex post-injury course with numerous sequelae that form part of a gradually evolving syndrome associated with chronic behavioral disturbances, seizure disorders, and protracted neurodegenerative diseases (Maas et al., 2008; Kovacs et al., 2014; Sharp et al., 2014). Reflecting the variable clinical presentation, TBI tissue pathology is highly inconsistent according to the type and severity of the injury and brain region involved (Sofroniew, 2009, 2015; Burda and Sofroniew, 2014). Due to the myriad and seemingly conflicting roles of astrocytes in the post-trauma environment, characterization of the contributions of heterogeneity and plasticity seems vital to the identification of targets for therapeutic manipulation both acutely for neuroprotective approaches and in the sub-acute/chronic period to enable for more effective axon regenerative approaches, which will continue to be the focus of extensive research efforts (Hilton et al., 2012; Cregg et al., 2014; Anderson et al., 2016; Geoffroy et al., 2016).

Several pre-clinical models of traumatic CNS injury have been utilized (Figure 3E; Jakeman et al., 2000; Metz et al., 2000; Dunham et al., 2010). Importantly, given the complexity of human traumatic injury, no one model alone can recreate all aspects of the injury process, therefore models are employed based on the study objectives and are thus limited in generalizability. In terms of injury mechanisms, SCI models can be broadly classified as contusion, crush, and transection (Hilton et al., 2013, 2019; Cheriyan et al., 2014), and recently emerging distraction and dislocation (Chen et al., 2016), with each mechanism having a unique tissue including glial response pattern. Contusion models are the most used due to their perceived clinical relevancy, although this relevancy has yet to be confirmed in a successful human trial of a neuroprotective treatment. The most common TBI models are controlled cortical impact (CCI) models and fluid percussion injury models, with the notable addition of models that mimic blast injuries or repeated mild TBI (i.e., concussion; Xiong et al., 2013). Differences in injury mechanism between the models and resultant secondary injury lead to differential astrocytic responses (Adams and Gallo, 2018; Boghdadi et al., 2020b), a consideration when attempting to generalize astrocyte responses across models.

### Role of Astrocytes in Traumatic Injury

Astrocytes (Sofroniew, 2020), OPCs (Hackett et al., 2016), and fibroblasts (Goritz et al., 2011; Soderblom et al., 2013; Dias and Göritz, 2018) have all been shown to contribute to the densely packed accumulation of cells in close proximity to injury epicenter following SCI and TBI, with continual modulation by microglia and infiltrating innate and adaptive immune cells (Silver and Miller, 2004; Silver, 2016; O’Shea et al., 2017; Bradbury and Burnside, 2019). For the purposes of this review, we focus on data pertaining to the astrocytic component (Faulkner et al., 2004; Anderson et al., 2018; Gu et al., 2019; Yang et al., 2020) but recognize that reactive astrogliosis is often accompanied by clusters or intermingling fibroblast-like cells and OPCs (Faulkner et al., 2004; Anderson et al., 2018; Dias et al., 2018; Gu et al., 2019; Yang et al., 2020). In agreement with a recent suggestion to avoid using the term “glial scar” or “scar” (Sofroniew, 2020), which should be reserved for mesenchymal or stromal scar tissue, we will refer to the previously termed “glial scar” as densely packed astrogliosis borders or densely packed reactive astrocytes. At the sub-acute/chronic traumatic injury epicenter (Figure 3F), there is a drastic loss of cells which overtime can result in the formation of a fluid-filled cyst surrounded by an inner layer of fibroblast-like cells and an outer layer of densely packed reactive astrocytes (Tran et al., 2021). Astrocytes are rapidly activated following traumatic injury culminating in the formation of a prominent densely packed astrogliosis border (Sofroniew, 2009; Adams and Gallo, 2018). Densely packed reactive astrocytes after TBI are partially regulated by monocyte invasion where reducing invasion using CCR2–/– mice results in increased astrocyte proliferation but perhaps surprisingly, decreased GFAP+ scar area, ECM deposition, and lesion size compared to controls (Frik et al., 2018). While traditionally viewed as a barrier to axon regeneration and thus functional recovery, the post-traumatic densely packed astrogliosis border is also ascribed a beneficial function in the mitigation of further damage to the surrounding spared tissue (Adams and Gallo, 2018). For example, attenuating densely packed astrogliosis through signal transducer and activator of transcription 3 (*stat3*) deletion results in increased inflammation, lesion volume, and reduced motor recovery compared to controls (Herrmann et al., 2008). Importantly, the role of the densely packed astrogliosis border appears to be dynamic throughout the post-injury period, reflecting the multiplicity of functions ascribed to it (Anderson et al., 2016; Adams and Gallo, 2018).

### Astrocyte Diversity in CNS Traumatic Injury

Diversity of reactive astrocytes following CNS trauma is thought to be driven largely by fluctuations in the inflammatory and cellular milieu of the post-injury environment (Hara et al., 2017; Adams and Gallo, 2018; Boghdadi et al., 2020b). RNA-seq performed on astrocytes 2 weeks following a spinal cord crush injury revealed differential expression of over 6,000 genes (Anderson et al., 2016), similar in magnitude with that observed post-ischemia (Zamanian et al., 2012; Anderson et al., 2016). Importantly, these reactive astrocytes demonstrate significant environmental-dependent plasticity (Hara et al., 2017; Boghdadi et al., 2020b). For example, integrin/N-cadherin-dependent collagen-1 signaling shapes astrocyte phenotypes in the post-injury spinal cord (Hara et al., 2017). Moreover, reactive astrocytes grafted into the spinal cord adopt an environmentally congruent phenotype (Hara et al., 2017). For example, reactive astrocytes grafted into the injured spinal cord retain the reactive phenotype, while those transplanted into the uninjured spinal cord revert phenotypically into resting astrocytes, confirmed by transcriptional analysis (Hara et al., 2017). After repetitive diffuse mild TBI, an atypical reactive astrocyte population was observed which featured a lack of GFAP expression, a downregulation of homeostatic proteins, and pronounced astrocyte coupling impairments, highlighting the importance of characterizing diversity across different types of injury (Shandra et al., 2019).

Extensive research has focused on manipulating the astrogliosis-associated deposition of growth-inhibitory ECM molecules (e.g., chondroitin sulfate proteoglycans; CSPGs) in the context of traumatic injury (McKeon et al., 1995; Ponath et al., 2018) in an attempt to improve axonal regeneration (Bradbury et al., 2002; Cregg et al., 2014; Burda et al., 2016; Tran et al., 2018). In a rat model of TBI, CSPG expression is increased in a subset of peri-lesional astrocytes, peaking at 7 days post-injury, which is temporally correlated with a reduction in peri-neuronal net density and increased GAP-43+ neurons (Harris et al., 2009, 2010; Yi et al., 2012). This is consistent with the observed increase in neural plasticity, which is an important intrinsic mechanism of functional recovery following trauma. Additionally, subsets of astrocytes were observed to express the complement component C3 as early as 3 days post-injury with further increase through 28 days post-injury, mainly clustered around the lesion core (Qian et al., 2019). Whether these C3+ astrocytes represent neurotoxic or pro-inflammatory phenotypes akin to “A1” astrocytes (Liddelow et al., 2017) remains to be determined. The potential role of these cells in axonal loss and retraction of transected axons from the lesion edge would be interesting to investigate as they remain key hurdles impeding regenerative and neuroprotective strategies in models of CNS trauma (Anderson et al., 2016; Geoffroy et al., 2016; Liddelow and Barres, 2016; Hilton et al., 2017).

### Potential for Astrocyte Plasticity Based on Disease-Associated Variables in Traumatic Injury

In the context of traumatic injury, we propose that astrocyte diversity across the disease-associated variables of time post-injury (i.e., temporal), distance from injury (i.e., topographical), age at which injury is sustained, and sex of the individual are examples where plasticity is likely to predominant over heterogeneity. Extensive work has been performed to characterize reactive astrogliosis in the context of SCI (Filous and Silver, 2016; Silver, 2016; Bradbury and Burnside, 2019), focusing predominately on temporal changes in GFAP and CSPGs expression and morphological changes. Importantly, there are limited peer-reviewed transcriptomics data available to date. scRNA-seq was used to generate transcriptomics data based on temporal changes post-injury after mouse contusion SCI at 1, 3, and 7 dpi (Milich et al., 2021). Gene Ontology (GO) Enrichment Analysis for differentially expressed genes performed on astrocytes at 1 dpi were “translation” and “biosynthetic processes,” while by 3 dpi astrocytes were defined by “mitochondrial function” and “oxidative phosphorylation,” and at 7 dpi astrocytes were related to “lipid processing” (Milich et al., 2021). Despite numerous remaining questions regarding the extent and functional relevance of temporal astrocyte diversity following CNS traumatic injury, it appears probable that dynamic changes are evident following injury with shifting roles in the evolving injury and recovery processes, hence representing plasticity in response to environmental fluctuations. Further elucidation with fate-mapping studies and functional interrogation is key to revealing effective means of manipulating these processes to promote neuroprotection and recovery following injury.

As with other CNS pathologies, the degree of reactive astrogliosis in traumatic injury is highly dependent on the distance from the lesion, secondary to the gradation of pathology radiating outwards from the lesion epicenter, as reflected by parallel gradients of axonal injury, vascular disruption, ischemia, and inflammation (Wanner et al., 2013; Okada et al., 2018; Boghdadi et al., 2020b). GFAP expression, CSPG expression, astrocyte proliferation, and astrocyte density is highest directly adjacent the lesion, with a decreasing taper with increasing distance (White et al., 2009; Wanner et al., 2013). Importantly, in the injured spinal cord there appears to be diversity amongst locally intermingled astrocytes equidistant to the lesion, including variable expression of GFAP, NESTIN, and brain lipid-binding protein (BLBP; White et al., 2009) and only certain astrocyte subsets proliferate and/or polarize in response to a cortical stab injury (Bardehle et al., 2013), which are predominately juxta-vascular, reflecting the influence of blood-borne elements (Bardehle et al., 2013). These findings highlight the complexity and extent of astrocyte diversity that exists in CNS trauma, largely a reflection of the complex post-injury environment, in keeping with our definition of plasticity.

Despite the increasing prevalence of aged SCI patients, there is limited data on reactive astrocyte diversity in the aged CNS. One study examined astrocytes in young (i.e., 4 month old) and aged mice (i.e., 18-month-old) at 1, 3, and 7 days after a controlled cortical impact TBI (Early et al., 2020). Histological analysis demonstrated a significant increase in GFAP+ area in the aged mice compared to young, but notably only at 7 dpi (Early et al., 2020). Generally, transcriptomics data performed on these astrocyte populations found disproportionate changes in genes associated with reactive astrocytes in the aged mice after TBI compared to their young counterparts, as might be expected based on findings from other pathologies and normal aging process. Importantly, these profiles were not aligned with classic “A1/A2” phenotypes, once again highlighting the existence of a spectrum of reactive astrocyte subsets rather than a binary classification. Given the increasing importance of age in clinical presentation of traumatic CNS injury and the significant astrogliosis observed in animal models in the chronic SCI setting (months to years after injury; Silver and Miller, 2004), further work is needed to better characterize reactive astrogliosis diversity in the aged CNS and determine the functional relevance of these changes to disease outcomes (Burda et al., 2016; Okada et al., 2018).

Males are more commonly affected by CNS trauma than females (Cripps et al., 2011; Devivo, 2012; Gupte et al., 2019), attributed to increased risk-related behaviors. Despite this, there is limited data on sex-related astrocyte diversity in CNS trauma. In fact, because of the bladder problems associated with SCI models, 71% of pre-clinical SCI models are performed in female mice (Stewart et al., 2020), whose shorter urethra eases the stress of bladder expressions. In murine models of severe TBI, females display a larger GFAP+ area compared to males in the first week after injury which resolves by 30 dpi (Villapol et al., 2017). Moreover, chemokine C-C motif Ligand 2 (CCL2) expression was reduced after cortical injury in female astrocytes as compared to their male counterparts, potentially reflecting altered immune cell recruitment (Acaz-Fonseca et al., 2015). In contrast, no sex-specific differences were noted in reactive astrocytes between male and female mice in a model of repetitive diffuse brain injury (Shandra et al., 2019), reflecting the importance of the specific model used. One of the main drivers of sex-specific differences is likely sex hormones, namely estrogen, which is in keeping with our definition of plasticity. The role of estrogens in astrocyte activation has been previously established (Arevalo et al., 2015) and recent data demonstrate that inhibiting estradiol synthesis promotes reactive astrogliosis in female mice after a controlled cortical impact TBI (Vierk et al., 2012), an effect not seen in males (Vierk et al., 2012). Therefore, the female CNS may be more sensitive to CNS trauma as compared to the male counterpart as was suggested previously (Vierk et al., 2012; Tabatadze et al., 2015; Bender et al., 2017). Further investigation of the influence of estrogens in models of CNS trauma is an important means of differentiation between hormonally driven effects (i.e., plasticity) and specific patterns of gene expression that are linked to sex chromosome codification (i.e., heterogeneity). 17β-estradiol has been shown to have neuroprotective effects in pre-clinical models of SCI and TBI (Siriphorn et al., 2012; Day et al., 2017), and therefore targeted manipulation of estrogen signaling pathways may be a viable therapeutic target.

### Potential for Astrocyte Heterogeneity Based on Disease-Associated Variables in Traumatic Injury

In contrast to plasticity, astrocyte heterogeneity in the context of traumatic injury may manifest as regionally variable responses to injuries at different locations across the neuraxis (i.e., brain vs. cervical spinal cord vs. thoracic spinal cord, etc.). Further characterization of astrocyte responses across models of CNS trauma utilizing fate-mapping and clonal analysis are important approaches to elucidate the extent of heterogeneity. For example, clonal analysis using the Star Track approach of proliferative astrocytes in a cortical stab injury model revealed a distinct progenitor cell origin as compared to non-proliferative astrocytes (Martín-López et al., 2013), consistent with our definition of heterogeneity. In the injured spinal cord, NSC-derived astrocytes more effectively constrain inflammation and the expansion of secondary damage (Sabelstrom et al., 2013), suggesting that distinct cellular origins may be a contributor to the diverse behavior of astrocytes observed following CNS trauma. Interestingly, a subset of reactive astrocytes in the post-trauma CNS displays certain characteristics of NSCs, including proliferative ability, multipotent lineage potential *in vitro*, and expression of several neurogenesis-associated genes (Buffo et al., 2008; Sirko et al., 2013). The potential for manipulation of this population has been demonstrated (Adams and Gallo, 2018; Magnusson et al., 2020; Zamboni et al., 2020) and represents a potentially attractive therapeutic approach to replace lost cell populations following trauma. Importantly, in models of CNS trauma there is also a potential contribution to observed diversity from astrocytes derived from other cells, including ependymal cells (Barnabé-Heider et al., 2010) and NG2 glia (Komitova et al., 2011; Hackett et al., 2016). For example, 2% of the astrocytes after SCI were lineage-traced to FOXJ1 ependymal cells, which was dependent upon injury proximity to the central canal (Ren et al., 2017). Fate mapping approaches also revealed that NG2+ cells can give rise to a small percentage of densely packed GFAP+ astrocytes, both after a cortical stab injury (Komitova et al., 2011) and contusive SCI (Hackett et al., 2016). Distinct functionality and transcriptomic differences have not yet been established for these identified cell populations, representing important areas for further research.

As for ischemic stroke and demyelination, additional fate-mapping data in models of CNS trauma to establish the extent of heterogeneity is required. As CNS trauma can occur at various locations across the CNS, regional differences in the astrocyte populations may have a significant impact on injury outcome, as well as the success of functional recovery. For example, diverse astrocyte populations may be a key determinant of the extent of primary and secondary injury, considering the finding that elevated GFAP protein levels in cerebral spinal fluid after SCI are correlated with both baseline injury severity and poorer neurological outcomes (Skinnider et al., 2021a). Furthermore, differences in the local neuronal populations and the interplay between astrocytes and neurons in the post-injury environment may influence the success of neural circuit remodeling and thus functional recovery clinically. Understanding of the contribution of heterogeneity across brain regions, injury types (e.g., contusion, crush, etc.), and across stages of the disease process (e.g., acute, sub-acute, chronic) is therefore critical. This can be accomplished with performance of fate-mapping studies combined with high-resolution transcriptomic approaches and functional assays to interrogate the functional relevance of identified astrocyte diversity in diverse models of CNS trauma (e.g., SCI, TBI, concussion, etc.).

## Perspectives on Astrocyte Diversity in CNS Disease

Effective treatments are lacking for many neurological pathologies, including stroke, MS, and CNS trauma, resulting in significant morbidity and mortality. As astrocytes serve multiple vital roles in the post-insult CNS, they represent key players in disease pathogenesis, as well as promising targets for therapeutic manipulation. Characterization of the extent and functional relevance of astrocyte diversity in conditions of CNS insult is likely to aid in this endeavor: however, given the complexity of astrocyte reactivity (Lukovic et al., 2015; Anderson et al., 2016; Liddelow and Barres, 2016; Silver, 2016; Escartin et al., 2021) this will require a significant research effort. In this review, we summarize the current state of knowledge of astrocyte diversity in three representative and clinically relevant CNS pathologies; ischemic stroke, demyelination, and traumatic injury, highlighting the need for further characterization and functional interrogation of the contributions of heterogeneity vs. plasticity across temporal, topographical, age-specific, and sex-specific variables as a foundation to guide future development of therapeutic treatments to mitigate the impact of these conditions.

### Areas of Future Research

Importantly, several knowledge gaps exist in the areas explored in this review. Most notably, there is a relative paucity of scRNA-seq studies characterizing astrocyte diversity in models of CNS trauma, as compared to demyelination and ischemic stroke. Moreover, as the vast majority of pre-clinical research has been performed in rodent models, increased use of non-human primate models or human tissue specimens is an important future avenue, as functional differences between species might pose a hurdle for clinical translation if not thoroughly addressed. Indeed, human astrocytes are known to be structurally more complex and diverse than rodent astrocytes (Oberheim et al., 2009; Zhang et al., 2016), as well as propagate Ca^2+^ waves approximately 4-fold faster than rodents in response to glutamate stimulation (Zhang et al., 2016), suggesting potential functional differences. Another critical area for future study is the influence of age on astrocyte diversity. This is particularly important not just for ischemic stroke, but for traumatic injury as well as older ages represent an increasing proportion of SCI and TBI, which is projected to increase in line with increasing age demographics in developed nations, and for progressive MS and the age-related decrease in remyelination efficiency. Considering that life expectancies of many of these clinical populations is increasing secondary to improved care and treatments, it will be essential to investigate astrocyte diversity after significant time periods after the onset of pathology (i.e., investigations in the 1–2 year post-injury chronic lesion sites within rodent models).

To meet these needs will require a combination of (1) increasing throughput of single-cell biology assays to achieve robust results across experimental conditions and biological replicates (Cao et al., 2020), (2) the application of multi-omic approaches to the context of CNS pathologies to avoid undue reliance on the functional interpretation of single-cell transcriptomes (Cao et al., 2018), and (3) bioinformatic methods development to aid biological investigators in differentiating cell type from cell state (Butler et al., 2018), to prioritize cell subsets most involved in the disease of interest (Skinnider et al., 2021b), and to avoid false positives that have the potential to drive research in unfruitful directions (Squair et al., 2021). Finally, these data will need to be met with stringent reporting standards to enable cross-disease investigations and to understand the conserved and differential responses of astrocytes across neurological disease.

In addition to conducting scRNA-seq-based characterization across multiple variables, it is increasingly important to further validate diverse astrocyte clusters in terms of origin and functionality, as most scRNA-seq analysis only provides a snapshot of gene expression within a specific context. Therefore, parallel assessments need to be performed to (1) validate mRNA and associated protein expression data, (2) establish the origin of identified clusters, and (3) assess functional differences. Furthermore, use of RNA-seq has the potential to identify cluster-specific, context-specific, or lineage-specific astrocyte markers across CNS states that will enable a more detailed interrogation of functionality and origin (e.g., with fate-mapping studies). New methods are also surfacing with the ability to predict ligand-targets links between interacting cells (Browaeys et al., 2020) and using RABID-seq facilitates the simultaneous investigation of the transcriptome of specific cells interacting with your cell type of interest (Clark et al., 2021) in disease modeling which could further be a powerful tool. In addition, recent tools, such as transposase-accessible chromatin profiling (sc-ATAC-seq; Jia et al., 2018) have the potential to yield information about how chromatin accessibility dictates astrocyte diversity. New technology is also paving the way for sequencing both genomic DNA and mRNA in the same cell which could facilitate a direct comparison of genomic diversity and transcriptomic diversity (Dey et al., 2015). Multi-omics is allowing for increasingly complicated analysis with the potential to, for example, compare genomics, transcriptomics, proteomics, and metabolomics in the same cells. In addition, considering the importance of mitochondrial dynamics in astrocytes (Jackson and Robinson, 2018), future work looking at mitochondrial DNA as a potential source of genomic diversity will likely be important. As the definition of heterogeneity hangs on the demonstration of distinct functionality, detailed assessments across identified astrocyte clusters focusing on a wide range of function-related outcome measures is essential (e.g., Ca^2+^ signaling, neurotransmitter uptake/buffering, inter-cellular connectivity, neurotrophic factor production, etc.; for comprehensive list, see Escartin et al., 2021). Further characterizations of potential functional differences in how astrocytes respond to neuronal activity through G-protein-coupled receptor signaling *in vivo* will likely prove worthwhile (Nagai et al., 2021a). Further areas of research should focus on metabolic profiling to interrogate other potential functional differences between clusters of interest and the continued use of conditional knockout mice for loss-of-functions studies. The other requirement for demonstrating heterogeneity is cells with distinct origins which will require sound fate mapping follow-up studies. Recent sequencing methods are providing more insight specific to tracking clones of cells across time (Weinreb et al., 2020) which likely yields a powerful tool for demonstrating the potential district origin of cell clusters.

### Astrocytes as Therapeutic Targets in CNS Disease

Astrocytes are promising therapeutic targets in several CNS diseases (Hamby and Sofroniew, 2010; Bradbury and Burnside, 2019; Valori et al., 2019). One potential approach is the use of viral vectors. Adeno-associated viruses (AAVs) demonstrate astrocyte-specific targeting in adult mice and non-human primates (Foust et al., 2009; Samaranch et al., 2012; Chan et al., 2017). Astrocyte-specific tropism may be increased through use of astrocyte-specific promoters (e.g., *Gfap*, *Aldh1L1*) and other viral capsid modifications (von Jonquieres et al., 2013; Meng et al., 2015; Vagner et al., 2016; Koh et al., 2017; Taschenberger et al., 2017). Lentiviral vectors pseudo-typed with glycoproteins from lymphocytic choriomeningitis virus (LCMV) or Moloney murine leukemia virus (MuLV) demonstrate astrocyte-specific targeting following intraparenchymal injection in rats (Cannon et al., 2011). Nanoparticles have also been used to target astrocytes (Zhou et al., 2018; Sharma et al., 2019), including the targeted regression of astrocyte-derived glioblastoma multiforme in mouse models (Jensen et al., 2013), inhibition of astrocyte-specific human immunodeficiency virus (HIV) replication *via* siRNA delivery (Gu et al., 2017), and astrocyte-specific delivery of mRNA *via* intraventricular administration (Tanaka et al., 2018). Alternatively, coupling of biologically active hydrophilic molecules (e.g., peptides, nucleic acids) to cell-permeable molecules can enable cell-specific delivery, as demonstrated in a mouse model of ALS (Martorana et al., 2012). Use of astrocyte-specific tropic factors, such as the phage AS1 homing peptide (Terashima et al., 2018) or herpes simplex virus type 1 (HSV-1) proteins (Valiante et al., 2015; Falanga et al., 2018) is a promising approach. Alternative approaches also include the use of Crispr-Cas9 gene-editing approach to specifically modify astrocytic genes (Huang and Nair, 2017; Kunze et al., 2018) and small molecules to modulate key signaling pathways (Zhang et al., 2015; Gao et al., 2017). As astrocytes are more resilient to the hostile post-injury environment in the CNS, direct cellular transplantation approaches may also be effective (Lepore et al., 2008; Izrael et al., 2018), although perhaps more limited in conditions, such as MS due to the disseminated nature of the lesions. Interestingly, dampening astrocyte reactivity (i.e., *via* STAT3 pathway inactivation) increases the number of OPC-derived Schwann cells in rodent demyelination lesions (Monteiro de Castro et al., 2015). In the context of the pronounced astrocyte loss seen in SCI, OPCs generate the majority of Schwann cells found in the contused lesion site (Assinck et al., 2017) and manipulating astrocytes to facilitate increased OPCs to differentiate into Schwann cells could be a promising alternative to invasive Schwann cell transplantation treatments which has been previously shown to promote repair and recovery in rat SCI models (Sparling et al., 2015; Assinck et al., 2020). Furthermore, astrocytes may represent an attractive source of cells for direct reprogramming to replace lost neurons or oligodendrocytes (Berninger et al., 2007; Heinrich et al., 2010). Forced expression of the transcription factors *Ngn2*, *Mash1*, or *Pax6* converts astrocytes to glutaminergic neuron-like cells in rodent models (Berninger et al., 2007), while overexpression of *Dlx2* or *Ascl1* converts them to GABAergic neuron-like cells (Heinrich et al., 2010; Liu et al., 2015). Further research is required to characterize the stability and functionality of these trans-differentiated “neurons,” but this is an intriguing approach. As transcriptional approaches and functional assessment further reveal the extent and relevance of astrocyte diversity in conditions of CNS disease there will undoubtedly be an expanded interest in astrocytes as therapeutic targets. Expansion of that knowledge is therefore critical to foster this development, specifically the identification of functionally relevant aspects of astrocyte diversity that are amendable to extrinsic manipulation (i.e., plasticity). This necessitates further research including expanded transcriptional studies, functional assays, and fate-mapping analyses to firmly establish and characterize this diversity.

### Astrocyte Diversity: An Interplay of Plasticity and Heterogeneity

**FIGURE 4:**
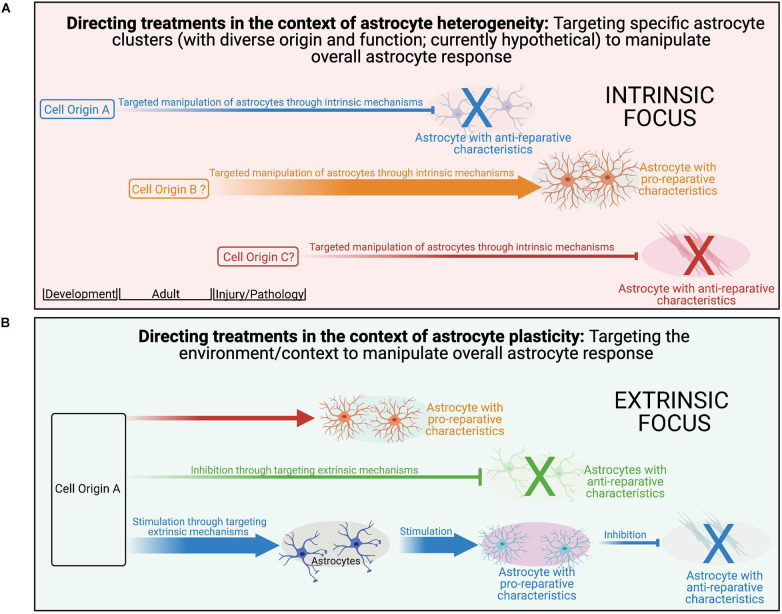
Distinguishing heterogeneity from plasticity is an important first step to ultimately direct treatments geared toward manipulating reactive astrogliosis at the right time and place through intrinsic mechanisms, extrinsic mechanisms, or both. **(A)** To the best of our knowledge, in the context of reactive astrogliosis and pathology, there are no perfect examples of astrocyte heterogeneity as defined in this review. It is likely, however, that further study will reveal evidence, as has been seen in other glial lineages. Identification of populations with distinct origins and unique functions will present opportunity for the therapeutic targeting of intrinsic pathways to manipulate the astrocyte response in the context of pathology. **(B)** Currently, with the multitude of data specific to astrocytes and their plasticity to local environmental changes, most evidence of astrocyte diversity in the context of pathology is suggested to fit into the plasticity category. Further research is needed to understand methods to manipulation the extinctic astrocyte environment to direct astrocytes in ways that will be beneficial in the context of pathology.

Using the framework proposed above, it remains unclear as to what proportion of the characterized astrocyte diversity in models of ischemic stroke, CNS demyelination, and traumatic injury represent heterogeneity vs. plasticity. We propose that without unequivocal demonstration of functional differences and clear fate-mapping data on the origin of the identified astrocyte clusters, most of the identified diversity to date likely proportionally represents plasticity in response to a dynamically changing injury environment. This is a point that has been argued for diversity in the oligodendrocyte lineage (Foerster et al., 2019) and most likely holds true for astrocytes. It is likely that combinations of both heterogeneity and plasticity exist across a non-exhaustive number of variables including spatial, temporal, age, and sex, reflecting the superimposition of plasticity in response to fluctuating and complex environments upon a background of developmentally pre-determined variance. This has significant therapeutic implications, as our definition of heterogeneity defines a population of astrocytes with a unique origin and function that may be more amendable to intrinsic manipulation approaches (Figure 4A). In contrast, plasticity implies a degree of malleability and thus would be amendable to extrinsic manipulation (Figure 4B). We propose here a simple model of intrinsic (e.g., viral targeting, DREADDs, optogenetics, transgenic manipulation, etc.) and extrinsic (e.g., manipulation of immune response, fibrotic scarring, ECM and stromal-derived matrix, exogenously administered cytokines, etc.) approaches for astrocyte-specific targeting, with the important caveat that extrinsic factors profoundly influence intrinsic mechanisms and no cell exists in complete isolation from their external environment, thus this distinction is not as clear-cut as presented here. Nonetheless, distinguishing heterogeneity from plasticity will yield a better understanding of the mechanisms of reactive astrogliosis and aspects of this response that can be targeted therapeutically. Given the significant interest in astrocytes as potential targets for therapeutic manipulation (Zhang and Barres, 2010; Schitine et al., 2015; Matias et al., 2019) and the recent increase in tools to assess cell diversity, astrocyte diversity in CNS pathologies remains an exciting and promising field of future research.

## Conclusion

Astrocytes display substantial diversity in ischemic stroke, CNS demyelination, and CNS traumatic injury, including temporal, topographical, sex-specific, and age-specific differences. Single-cell transcriptional approaches have significantly expanded our knowledge of the extent of this diversity, in the process altering our view of reactive astrocytes from a binary categorization to that of a spectrum of nuanced activation states involving hundreds of differentially expressed genes. Despite this, there are notable gaps in our knowledge, most notably whether this identified diversity represents heterogeneity or plasticity. As discussed throughout the review, the combination of transcriptomics approaches to further characterize the extent of diversity present with parallel functional assessments and expanded fate-mapping studies are critical areas of future study to fully develop our understanding of this diversity and to guide development of therapeutic approaches aimed at mitigating the morbidity and mortality of those afflicted with these conditions.

## Author Contributions

AJM, RJMF, WT, and PA contributed to the conception of review. AJM and PA contributed to design, original drafting, and figure preparation of the review. AJM, JWS, RJMF, WT, and PA contributed to writing, editing, and approval of final manuscript. All authors contributed to the article and approved the submitted version.

## Conflict of Interest

The authors declare that the research was conducted in the absence of any commercial or financial relationships that could be construed as a potential conflict of interest.

## Publisher’s Note

All claims expressed in this article are solely those of the authors and do not necessarily represent those of their affiliated organizations, or those of the publisher, the editors and the reviewers. Any product that may be evaluated in this article, or claim that may be made by its manufacturer, is not guaranteed or endorsed by the publisher.
